# Protein arginine methyltransferases in cancer: mechanisms, functions, and therapeutic opportunities

**DOI:** 10.1186/s12929-026-01240-3

**Published:** 2026-04-02

**Authors:** Yoonae Jeong, Yena Cho, Yong Kee Kim

**Affiliations:** 1 College of Pharmacy, Sookmyung Women’s University, Seoul, 04310 Republic of Korea; 2 Muscle Physiome Research Center and Research Institute of Pharmaceutical Sciences, Sookmyung Women’s University, Seoul, 04310 Republic of Korea

**Keywords:** Post-translational modification, PRMTs, Arginine methylation, Epigenetic regulation, Metabolic reprogramming, Cancer therapy

## Abstract

Protein arginine methyltransferases (PRMTs) catalyze the methylation of arginine residues on both histone and non-histone substrates, orchestrating cellular processes such as transcriptional regulation, RNA splicing, signal transduction, and DNA damage response. Because dysregulated methylation reprograms epigenetic and post-transcriptional landscapes to promote malignant transformation, aberrant PRMT activity is closely associated with tumorigenesis and cancer progression. Major family members, containing PRMT1, CARM1, PRMT5, and PRMT6, regulate gene expression through site-specific histone methylation, thereby contributing to the transcriptional activation or repression. PRMTs also methylate a wide range of non-histone proteins, including transcription factors, splicing regulators, and signaling intermediates, to coordinate cell cycle progression, DNA repair, and RNA metabolism. Collectively, PRMT-mediated methylation contributes to higher-order cancer phenotypes, including metabolic reprogramming―through modulation of glycolytic flux, lipid biosynthesis, and redox homeostasis―and immune evasion via altered immune signaling and checkpoint pathways within the tumor microenvironment. Recent advances in chemical biology have led to the development of selective PRMT inhibitors, several of which are currently under clinical evaluation. In this review, we provide a comprehensive and integrative overview of PRMT biology, systematically organizing current knowledge from multilayered regulatory mechanisms to downstream oncogenic effects and emerging therapeutic opportunities.

## Background

Post-translational modifications (PTMs) are covalent chemical alterations that regulate protein activity, stability, subcellular localization, and intermolecular interactions [1]. Well-established modifications, including phosphorylation, acetylation, methylation, and emerging modifications such as lactylation and succinylation, coordinate essential cellular processes [2, 3]. Dysregulation of PTM networks is now recognized as a key driver of cancer development, reshaping signaling cascades, metabolic programs, and immune responses through complex regulatory crosstalk [1, 4].

Among these modifications, protein arginine methylation plays an integrative role by linking chromatin-associated gene regulation with cytoplasmic signaling pathways. Moreover, protein arginine methyltransferases (PRMTs) have emerged as a central epigenetic and signaling regulator in tumor biology. By modifying both histone and non-histone substrates, PRMTs regulate transcriptional programs, RNA processing, DNA damage responses, and signal transduction pathways [5]. Aberrant PRMT activity disrupts these multilayered regulatory mechanisms, contributing to hallmark oncogenic processes such as genomic instability, metabolic reprogramming, and immune evasion within the tumor microenvironment [6, 7]. Given these multifaceted oncogenic roles, PRMTs are under active investigation as therapeutic targets, with several selective inhibitors currently advancing through preclinical and clinical development [8, 9]. Moreover, altered PRMT expression and substrate methylation patterns are frequently associated with adverse clinical outcomes, highlighting their potential as prognostic biomarkers [10].

In this review, we summarize the current insights into PRMT-mediated arginine methylation in cancer biology, focusing on its oncogenic functions, therapeutic implications, and recent advances in PRMT-targeting strategies. We further discuss ongoing efforts in pharmacological modulation and combination therapies that may open new avenues for precision oncology.

## A short history of PRMT research

**Fig. 1:**
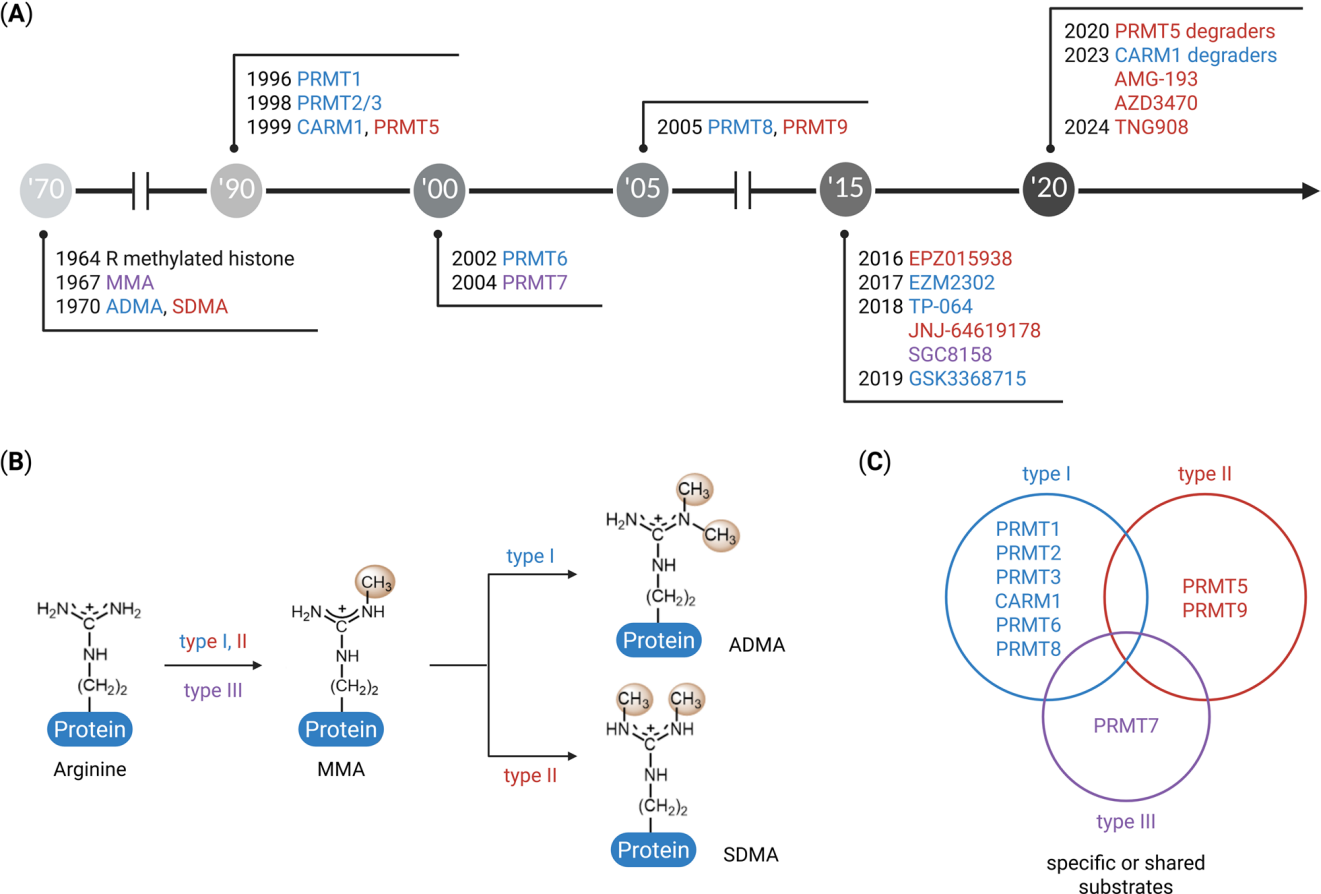
Timeline and classification of PRMTs. **A** Chronological timeline highlighting major milestones in PRMT research, including the first reports of arginine methylation and the identification of PRMT family members. Key developments in PRMT inhibitors and degraders are also indicated. Blue represents type I PRMTs and their inhibitors, red represents type II PRMTs and their inhibitors, and purple represents the type III PRMT and its inhibitor. **B** Schematic of arginine methylation reactions. PRMTs catalyze the transfer of methyl groups from S-adenosylmethionine (SAM) to the guanidino group of protein arginine residues. Type I enzymes generate asymmetric dimethylarginine (ADMA), type II enzymes generate symmetric dimethylarginine (SDMA), and type III enzymes catalyze monomethylation (MMA). **C** Venn diagram showing classification of PRMT family members based on their methylation type and substrate specificity. Overlapping regions indicate PRMTs that can act on shared substrates

The earliest evidence of arginine methylation emerged in the mid-twentieth century when Allfrey et al. (1964) reported methylated arginine and lysine residues in histones, suggesting their roles in gene regulation (Fig. 1A) [11]. Shortly thereafter, Paik and Kim used ^14^C-labeled S-adenosylmethionine (SAM) in calf thymus histones to detect novel methylated amino acids, leading to the identification of monomethyl arginine (MMA) and the first arginine methyltransferase [12, 13]. Subsequently, all three methylated arginine species, including asymmetric dimethylarginine (ADMA) and symmetric dimethylarginine (SDMA), were identified in human urine [14] and calf brain proteins [15]. During the same period, arginine methylation of myelin basic proteins was reported [16, 17], demonstrating that this modification extends beyond histones. In the 1980s, research focused on enzymes that catalyze arginine methylation. Kim et al. (1988) partially purified two methyltransferases from the bovine brain, one acting on histones and the other on myelin [18]. This histone-directed enzyme was later shown to methylate heterogeneous nuclear ribonucleoproteins (hnRNPs) [19], linking arginine methylation to RNA processing and metabolism.

A breakthrough occurred in 1996 with the cloning of mammalian PRMT1 and its yeast homolog, Hmt1/Rmt1, which established the evolutionary conservation of arginine methylation [20]. Between 1998 and 2001, the canonical PRMT family expanded rapidly. PRMT2 (1998) was identified as a PRMT1-associated factor [21], PRMT3 (1998) was identified via yeast two-hybrid screening [22], PRMT4/CARM1 (1999) was identified as a coactivator-associated PRMT [23], and PRMT5 (1999) was identified as a JAK2-interacting protein with histone methylation activity [24]. Subsequent studies identified PRMT6 (2002) [25], PRMT7 (2004) [26], and PRMT8 (2005) [27]. In 2005, a candidate gene later designated PRMT9 was proposed based on genomic analysis [27], and its enzymatic activity was subsequently characterized in later study [28]. Together, these findings established the nine-member family, whose members exhibit distinct substrate specificities, subcellular localizations, and biological functions. Based on their catalytic activity, PRMTs are classified into three enzymatic subtypes: type I PRMTs (PRMT1, 2, 3, 4, 6, and 8) sequentially convert arginine to MMA and then to ADMA; type II PRMTs (PRMT5 and 9) generate MMA and subsequently SDMA; type III PRMTs (PRMT7) catalyze only MMA formation (Fig. 1B). Although each PRMT exhibits substrate preference, certain substrates can be shared among multiple family members (Fig. 1C). Advances in X-ray crystallography and cryoelectron microscopy have revealed detailed PRMT structures, providing insights into their catalytic mechanisms and substrate recognition [29, 30]. Proteomics-based studies have uncovered numerous PRMT substrates, establishing their broad regulatory roles in transcription, RNA processing, DNA repair, cell cycle regulation, metabolism, and immune modulation [5, 31]. Therefore, dysregulated PRMT expression and activity is linked to oncogenic signaling outputs and malignant phenotypes.

The recognition of PRMTs as therapeutic targets has accelerated the development of small-molecule inhibitors of PRMTs (Fig. 1A). The first PRMT5 inhibitor, EPZ015938 (GSK3326595; NCT02783300), entered Phase I clinical trials in 2016, followed by JNJ-64619178 (2018) (NCT03573310) and GSK3368715 (NCT03666988), the first PRMT1 inhibitor (2019). Although the mechanism of action of these agents has been clinically established, their therapeutic windows and antitumor activities are limited. Recently, next-generation PRMT5 inhibitors such as AMG-193 (NCT05975073) and AZD3470 (NCT06130553, NCT06137144), were clinically evaluated in 2023 by leveraging methylthioadenosine (MTA)-cooperative binding mechanisms to enhance the selectivity for methylthioadenosine phosphorylase (MTAP)-deleted tumors. These advances mark a transition from enzymology to translational oncology, positioning PRMTs as a new class of druggable targets for cancer therapy development.

## The PRMT family: structure, catalytic diversity, and regulation

### Classification and structural features of PRMTs

**Fig. 2:**
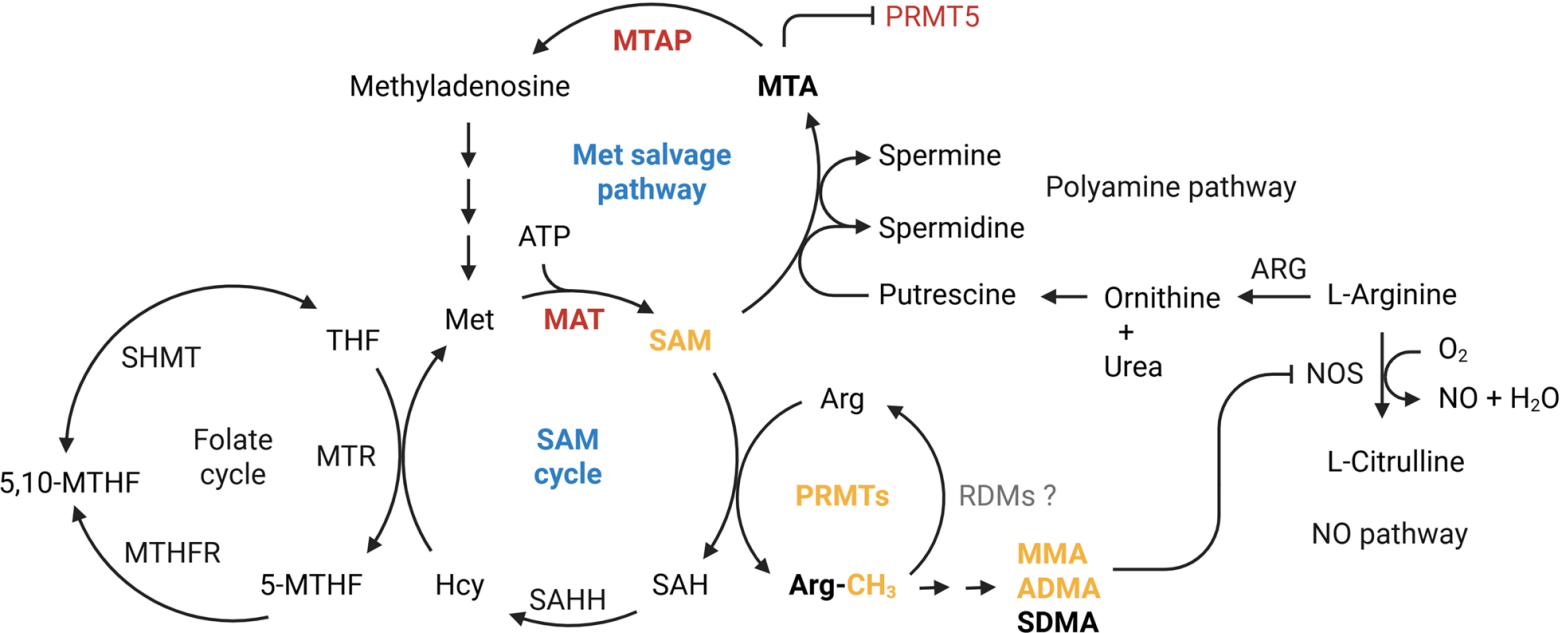
S-adenosylmethionine metabolism and protein arginine methylation. S-adenosylmethionine (SAM), a central methyl donor, is synthesized from methionine and ATP by methionine adenosyltransferase (MAT) in the SAM cycle. SAM-dependent methylation by PRMTs generates MMA, ADMA, and SDMA, along with S-adenosyl homocysteine (SAH), which is converted to homocysteine and recycled to methionine through the folate cycle (involving SHMT, MTHFR, and MTR). SAM is also consumed in the polyamine biosynthetic pathway to form spermidine and spermine, which produce methylthioadenosine (MTA). MTA is recycled to methionine through the MTAP-dependent methionine salvage pathway; however, MTAP is frequently deleted in cancer, resulting in MTA accumulation. Arginine metabolism intersects these pathways: NOS converts L-arginine to nitric oxide and citrulline, whereas arginase produces ornithine and urea. The breakdown of methylated proteins releases MMA and ADMA, which are endogenous NOS inhibitors associated with cardiovascular risk

**Fig. 3:**
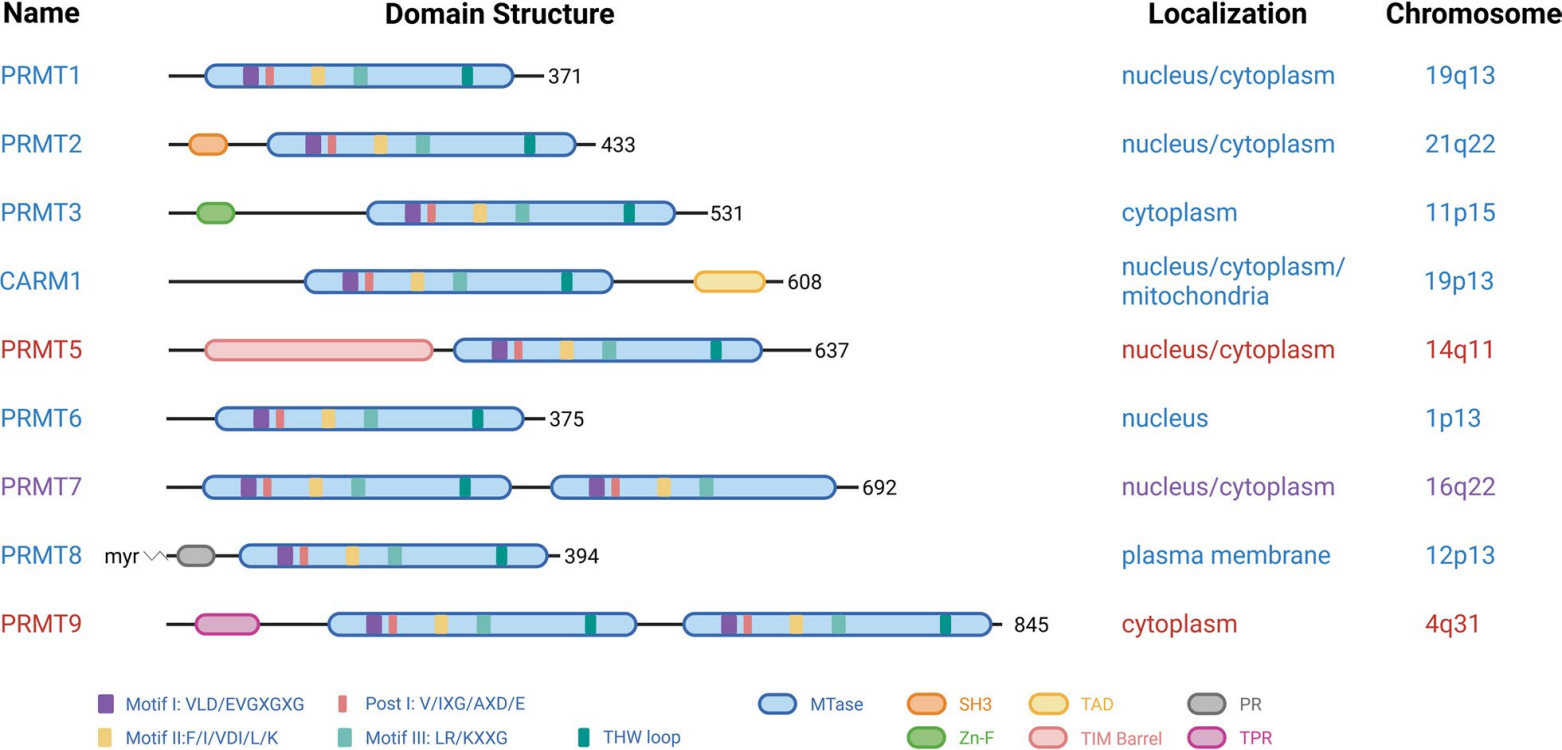
Classification and structural domains of mammalian PRMTs. Classification and structural domains of mammalian PRMTs. Nine PRMTs have been identified, each containing conserved motifs important for catalytic activity, including Motif I (VLD/EVGXGXG), Post-I (V/IXG/AXD/E), Motif II (F/I/VDI/L/K), Motif III (LR/KXXG), and the THW loop. PRMTs are classified by enzymatic type (I, II, and III) and show distinct subcellular localization

Protein methylation reactions, including arginine methylation, require SAM as a universal methyl donor. Intracellular SAM availability is tightly controlled by the SAM cycle and methionine salvage pathway, in which methionine is converted to SAM by methionine adenosyltransferase (MAT) (Fig. 2) [32]. Protein arginine methylation is catalyzed by PRMTs, which transfer a methyl group from SAM to the guanidino nitrogen of arginine residues [33]. Nine human PRMTs (PRMT1–PRMT9) have been identified, each encoded by a distinct chromosomal locus. All PRMTs share a conserved catalytic Rossmann-fold domain required for SAM binding and catalysis, comprising four consensus motifs: Motif I (VLD/EVGXGXG), post-I (V/IXG/AXD/E), Motif II (F/I/VDI/L/K), and Motif III (LR/KXXG), along with a THW loop that facilitates methyl transfer. The N- and C-terminal extensions confer substrate specificity, localization, and cofactor interactions. Notably, CARM1 possesses an extended C-terminal transactivation domain (TAD) responsible for its transcriptional coactivator function, distinguishing it from other PRMTs (Fig. 3). These distinct methylation patterns critically shape protein–protein and protein–RNA interactions, influencing diverse biological processes [5].

Beyond catalytic specificity, PRMTs exhibit distinct substrate sequence preferences that define their biological context. Most type I and II PRMTs target arginine–glycine (RG/RGG) rich motifs, which are common in RNA-binding and chromatin-associated proteins. In contrast, CARM1 preferentially recognizes proline–glycine–methionine (PGM) motifs found in transcriptional coactivators and splicing factors, whereas PRMT7 targets RxR motifs enriched in stress response proteins [5, 31, 32]. These sequence preferences, along with interactions with adaptor proteins and PTMs, ensure the context-dependent regulation of PRMT activity and substrate selectivity.

### Molecular and biophysical consequences of arginine methylation

While catalytic specificity and sequence preference determine where methylation occurs, the biological outcome ultimately depends on how methylation alters the physicochemical properties of arginine residues. At the molecular level, arginine methylation regulates protein structure and function by chemically remodeling the guanidinium group while largely preserving its positive charge. The addition of methyl groups increases steric bulk, reduces hydrogen-bond donor capacity, and enhances local hydrophobicity [5, 32]. These changes reshape electrostatic, hydrogen-bonding, and cation–π interactions, thereby subtly modulating intra-molecular contacts that influence folding stability and conformational flexibility, while reconfiguring inter-molecular interfaces with acidic proteins, nucleic acids, and membrane surfaces [34]. Such effects are particularly pronounced within intrinsically disordered regions, especially RG/RGG motifs. In these regions, methylation shifts conformational ensembles and modulates multivalent interaction networks that drive liquid–liquid phase separation, thereby regulating the formation, stability, and material properties of membraneless organelles [35–37]. In addition, methylated arginine residues function as selective docking sites for specialized reader domains, including Tudor-containing proteins, while sterically hindering alternative binding events [38–40]. Through this coordinated capacity to create, redirect, or block interaction surfaces, arginine methylation operates as a dynamic molecular rheostat that fine-tunes binding specificity, complex assembly, subcellular localization, and enzymatic activity without necessitating large-scale structural rearrangements.

### Multilayered regulation of PRMT activity

PRMTs are regulated through multilayered mechanisms that integrate signaling pathways, metabolic inputs, and protein–protein interactions. For example, CARM1 is tightly regulated by coordinated PTMs and subcellular localization that dynamically shape its catalytic output. CARM1 undergoes automethylation at R551 within its C-terminal region, a modification required for its full transcriptional activity and regulation of pre-mRNA splicing [41]. In addition, CARM1 is phosphorylated during mitosis, altering its enzymatic activity and chromatin association, thereby linking arginine methylation to cell cycle progression [42–44]. Ubiquitination of CARM1 has also been reported to influence its stability and proteasomal turnover, adjusting enzyme abundance in response to energy stress [45]. Furthermore, alternative splicing generates CARM1 isoforms with distinct catalytic properties and substrate selectivity, adding another layer of regulation [46–48]. In parallel, PRMT5 regulation is largely governed by its obligate complex formation and PTMs. PRMT5 requires association with its obligate cofactor MEP50 to achieve full catalytic activity and proper substrate recognition [46–48]. Tyrosine phosphorylation of PRMT5 by upstream kinases inhibits its enzymatic activity [49, 50], while K63-linked ubiquitination of PRMT5 promotes interaction with MEP50, leading to an increase in enzyme activity [51]. PRMT5 activity is also sensitive to intracellular levels of SAM, linking its function to cellular metabolic status [52, 53]. In cancer contexts, oncogenic signaling pathways often upregulate PRMT5 expression or enhance its complex assembly, thereby promoting symmetric dimethylation of histones and non-histone substrates involved in proliferation and RNA splicing [5, 54]. Collectively, these examples illustrate that PRMT regulation occurs at multiple levels: (i) dynamic PTMs such as automethylation, phosphorylation, and ubiquitination that modulate catalytic output and stability, (ii) isoform control, (iii) cofactor-dependent complex assembly, (iv) metabolic control via SAM availability, and (v) transcriptional control. Such multilayered regulation ensures that PRMT activity is precisely tuned in a context-dependent manner in both normal physiology and disease states.

### Emerging evidence for arginine demethylation

Although arginine methylation has long been considered an irreversible modification, recent studies have suggested that certain JmjC domain–containing proteins may possess arginine demethylase (RDM) activity toward both histone and non-histone substrates [55]. For example, KDM3B has been reported to demethylate H4R3me2s [56], whereas Mina53 has been proposed to target H4R3me2a and p53 arginine methylation [57]. In addition, KDM5C has been implicated in regulating ULK1 arginine methylation [58], and KDM4A erases H3R17me2a [59] and also removes arginine methylation of PI3KC2α and IDH2 [60, 61]. Collectively, these findings indicate that arginine methyl marks deposited by PRMTs could be enzymatically reversible under specific cellular contexts, linking arginine demethylation to processes such as transcriptional regulation, tumor progression, autophagy, and mitotic control. Nevertheless, the substrate specificity, catalytic efficiency, and physiological relevance of these demethylation events remain incompletely understood, and further biochemical and structural validation will be essential to establish RDM as a broadly operative regulatory mechanism.

## Oncogenic roles of PRMTs in cancer

Aberrant expression and activity of PRMTs have been increasingly recognized as driving forces of tumorigenesis. PRMTs influence nearly every hallmark of cancer through their diverse substrates and cellular functions, including sustained proliferation, metabolic reprogramming, evasion of apoptosis, enhanced migration and invasion, and resistance to therapy. Mechanistically, PRMTs integrate epigenetic, transcriptional, post-transcriptional, and signaling networks to reprogram the oncogenic state of the cell. The role of each PRMT in cancer is summarized in Table 1.

**Table 1:** The role of PRMTs in cancer

PRMTs	Cancer type	Expression	Function	Biological mechanism	Refs
PRMT1					
	Breast cancer	High	Oncogenic	ERα methylation (R260) activates IGF-1 signaling	[257]
				EZH2 methylation (R342) stabilizes EZH2 and promotes EMT/metastasis	[91]
				C/EBPα methylation (R35/156/165) promotes cyclin D1 expression and cell proliferation	[83]
				H4R3me2a at ZEB1 promoter promotes EMT, metastasis, and regulates senescence	[70]
				SRSF1 methylation (R93, R97 and R109) promotes exon inclusion and cell proliferation	[100]
				DDX3 methylation stabilizes DDX3, coordinating mitochondrial homeostasis to promote metastasis	[259]
	Pancreatic cancer	High	Oncogenic	Gli1 methylation (R597) promotes transcriptional activity and its oncogenic functions	[260]
				HSP70 methylation (R416, R447) stabilizes *BCL2* mRNA, promoting apoptosis resistance	[261]
				PRMT1 regulates RNA metabolism and DNA damage response, promoting PDAC growth	[107]
		-	Tumor-suppressive	p14^ARF^ methylation (R96/R99) triggers stress-induced apoptosis via release from nucleolus	[262]
	Colorectal cancer	High	Oncogenic	EGFR methylation (R198/R200) enhances EGF signaling and cetuximab resistance	[111]
				H4R3me2a recruits SMARCA4 to activate EGFR/TNS4 signaling, promoting cancer progression	[69]
				NONO methylation (R251) promotes colorectal cancer growth and metastasis	[170]
	Gastric cancer	High	Oncogenic	cGAS methylation (R133) suppresses cGAS/STING signaling and anti-tumor immunity	[208]
				PRMT1 activates β-catenin signaling via MLXIP recruitment, promoting gastric cancer metastasis	[84]
				c-Fos methylation (R287) stabilizes c-Fos, activates AP-1, and promotes gastric cancer progression	[262]
	Lung cancer	High	Oncogenic	Twist1 methylation (R34) promotes EMT and lung cancer metastasis	[263]
	HCC	High	Oncogenic	PRMT1 promotes cell proliferation and survival, serving as a prognostic marker and therapeutic target	[264]
				PHGDH methylation (R236) enhances serine synthesis and promotes HCC proliferation	[190]
	ccRCC	High	Oncogenic	PRMT1 regulates RNA metabolism; its inhibition induces R-loops and DNA damage	[108]
				PRMT1 promotes ccRCC growth and drug resistance via LCN2-Akt-RB signaling	[265]
	Retinoblastoma	High	Oncogenic	PRMT1 promotes retinoblastoma proliferation via p53/p21/CDC2/Cyclin B signaling	[133]
	Melanoma	High	Oncogenic	PRMT1 methylates/activates ALCAM, promoting melanoma cell growth and metastasis	[266]
	Head and neck cancer	High	Oncogenic	PRMT1 promotes HNC growth and migration via ADMA-mediated protein methylation	[267]
	ESCC	High	Oncogenic	PRMT1 promotes ESCC progression via activation of Hedgehog signaling	[268]
	NPC (EBV-associated)	-	Oncogenic	PRMT1 methylates PGC-1α, stabilized by EBV LMP1, promoting PD-L1-mediated immune escape	[175]
				PRMT1 maintains ESCC tumor-initiating cells via H4R3me2a, activating Wnt/Notch signaling	[269]
	AMKL	High	Oncogenic	PRMT1 drives AMKL growth by boosting glycolysis and inhibiting fatty acid oxidation	[181]
PRMT2					
	Breast cancer	High	Oncogenic	PRMT2/variants enhance ERα signaling to promote breast cancer cell proliferation	[270]
		Low	Tumor-suppressive	PRMT2 inhibits ERα/AP-1-mediated cyclin D1 transcription, suppressing cancer cell proliferation	[263]
	Colorectal cancer	High	Oncogenic	PRMT2 promotes CRC progression and immune suppression via H3R8me2a at *WNT5A* promoter	[72]
	RCC	High	Oncogenic	PRMT2 drives RCC progression by activating Wnt signaling via H3R8me2a at *WNT5A* promoter	[73]
	Glioblastoma	High	Oncogenic	PRMT2 drives GBM progression by maintaining oncogenic transcription via H3R8me2a	[71]
PRMT3					
	Breast Cancer	High	Oncogenic	H4R3me2a-mediated activation of ER stress signaling promotes proliferation and metastasis	[171]
	Glioblastoma	High	Oncogenic	PRMT3 promotes GBM progression by enhancing HIF1A and glycolytic metabolism	[185]
	Pancreatic cancer	High	Oncogenic	PRMT3 drives pancreatic cancer growth via GAPDH methylation (R248)-mediated metabolic rewiring	[183]
CARM1					
	Breast cancer	High	Oncogenic	CARM1 promotes *CCNE1* transcription via H3R17/R26 methylation, promoting cell cycle progression	[127]
				BAF155 methylation (R1064) drives metastasis via regulation of oncogenic chromatin programs	[92, 93]
				LSD1 R838 methylation drives metastasis via epigenetic regulation of E-cadherin and vimentin	[159]
		-	Tumor-suppressive	CARM1 coactivates ERα to induce differentiation and suppress proliferation in ERα-positive cancer	[271]
				MED12 methylation (R1862/R1912) enhances chemotherapy sensitivity in breast cancer	[94, 271]
	Lung cancer (SCLC)			ESRP1 reverses SCLC chemoresistance by regulating CARM1 splicing and inhibiting EMT	[166]
	Colorectal cancer	High	Oncogenic	CARM1 promotes CRC by enhancing β-catenin-mediated transcription through H3R17me2a	[116]
	Gastric cancer	High	Oncogenic	H3R17me2-mediated G6PD expression and PPP promote gastric cancer cell survival low glucose	[194]
	Pancreatic cancer	Low	Tumor-suppressive	MDH1 methylation (R230) suppresses glutamine metabolism and regulate redox homeostasis	[193]
	HCC	Low	Tumor-suppressive	GAPDH methylation (R234) suppresses glycolysis and delays proliferation in liver cancer cells	[187]
		High	Oncogenic	CARM1 drives HCC progression by activating Akt/mTOR pathway, enhancing migration and invasion	[272]
	Ovarian cancer	High	Oncogenic	CARM1 promotes EZH2-dependent silencing of tumor suppressor genes	[273]
	AML	High	Oncogenic	RUNX1 methylation (R223) blocks myeloid differentiation in AML	[85]
				CARM1 drives AML by promoting proliferation and blocking differentiation	[274]
PRMT5					
	Lymphoma	High	Oncogenic	PRMT5 drives lymphoma cell proliferation through Wnt/β-catenin activation via H3R8me2s	[167]
	DLBCL	High	Oncogenic	PRMT5 drives DLBCL proliferation via BCR-induced PI3K–Akt and NF-κB signaling	[275]
	Leukemia/lymphoma	High	Oncogenic	PRMT5 represses tumor suppressor genes via H3R8/H4R3 hypermethylation	[276]
	AML	-	Oncogenic	SRSF1 methylation (R93, R97, R109) regulates alternative splicing of essential genes	[101]
		-	Oncogenic	PRMT5 promotes AML via H4R3me2s-mediated miR-29b silencing, leading to Sp1/FLT3 activation	[76]
	Breast cancer	High	Oncogenic	ZNF326 methylation (R175) regulates alternative splicing and mRNA stability	[102]
				PRMT5 promotes stemness and doxorubicin resistance by regulating OCT4, KLF4, and MYC	[277]
				PRMT5 interacts with TRAF4 to activate NF-κB signaling, promoting breast cancer proliferation	[278]
				PRMT5 regulates breast cancer stem cell function via histone methylation and FOXP1 expression	[78]
				PRMT5 scaffolds GR to promote glucocorticoid-induced transcription and cell migration in TNBC	[173]
				Repression of E-cadherin via H4R3me2s and activation of vimentin via H3R2me2s	[162]
	Ovarian cancer	High	Oncogenic	PRMT5 regulates tumor cell growth and apoptosis dependent on E2F-1	[131]
				ENO1 methylation (R9me2s) promotes dimerization and enhances glycolysis	[279]
	Cervical cancer	High	Oncogenic	PRMT5 drives cervical cancer metastasis via the Snail/PRMT5/NuRD complex-mediated EMT	[161]
	Lung cancer	High	Oncogenic	PRMT5 promotes lung cancer growth and metastasis via the H4R3me2s–miR-99–FGFR3 axis	[280]
				PRMT5 promotes lung cancer cell proliferation by directly interacting and activating Akt	[281]
				PRMT5-SHARPIN complex-mediated H3R2me1 activates transcription of metastasis-related genes	[282]
				ENO-1 methylation (R50) enhances its localization to the surface membrane	[283]
				H4R3me2s deposition on *CD274* promoter represses PD-L1 expression	[210]
				PRMT5 dimethylates at R41 and stabilizes KLF5 to activate Akt/GSK3β pathway	[284]
	Prostate cancer	High	Oncogenic	PRMT5 recruits pICln to methylate H4R3 at AR promoter, activating AR/AR-V7 transcription	[285]
				AR methylation (R761) suppresses differentiation gene expression and promoting proliferation	[286]
	Gastric cancer	High	Oncogenic	PRMT5 is upregulated in gastric cancer, enhances proliferation, invasion, and migration	[287]
				PRMT5 binds c-Myc to repress tumor suppressor genes via H4R3me2s	[86]
				PRMT5-mediated histone methylation recruits DNMT3A to silence *IRX1*	[172]
	HCC	High	Oncogenic	PRMT5 is overexpressed in HCC and colon cancer, promotes invasiveness via MMP-2 upregulation	[288]
				PRMT5 promotes HCC proliferation by activating ERK signaling and suppressing BTG2	[289]
				Metadherin-PRMT5 complex enhances metastasis through Wnt-β-catenin pathway	[117]
	Pancreatic cancer	High	Oncogenic	PRMT5-mediated epigenetic silencing of FBW7 stabilizes c-Myc at the protein level	[290]
				PRMT5 promotes EMT through activation of the EGFR/Akt/β-catenin signaling pathway	[113]
	Colorectal cancer	High	Oncogenic	YBX1 Methylation (R205) is essential for NF-κB activation and CRC growth and migration	[125]
				PRMT5 cooperates with EZH2 to epigenetically silence *CDKN2B*, promoting CRC progression	[77]
				PRMT5 activates the EGFR/Akt/GSK3β signaling pathway, promoting CRC proliferation	[291]
				SMAD4 methylation (R361) activates TGF-β signaling and promotes metastasis	[115]
				ZEB2 recruits TWIST1, PRMT5, and NuRD to epigenetically silence E-cadherin	[163]
	Melanoma	High	Oncogenic	SHARPIN facilitates PRMT5 activity that increases SOX10 and PAX3 expression	[292]
				Regulation of *MDM4* expression via alternative splicing, resulting in resistance to CDK4/6 inhibitor	[97]
	ESCC	-	Oncogenic	MTHFD1 methylation (R173) enhances NADPH production, promoting metastasis	[176]
	Glioblastoma	High	Oncogenic	SWI/SNF-associated PRMT5 generates H3R8me2s to repress ST7 and NM23	[293]
				PRMT5 regulates splicing and stemness in glioblastoma	[99]
				PRMT5 regulates PTEN/Akt/ERK signaling to maintain both differentiated and stem-like tumor cell	[294]
	Neuroblastoma	High	Oncogenic	Akt1 methylation (R15) promotes tumor metastasis	[120]
	Bladder cancer	High	Oncogenic	PRMT5 activates NF-κB signaling and upregulates anti-apoptotic genes *BCL-XL/cIAP1*	[126]
	MTAP-deleted cancer			Increased endogenous MTA inhibits PRMT5 activity and induces vulnerability toward PRMT5	[247, 295]
PRMT6					
	Breast cancer	High	Oncogenic	PRMT6/PARP1/CRL4B forms transcriptional-repression complex and promotes metastasis	[174]
				STAT3 methylation (R729) promotes its membrane localization and promotes cancer cell metastasis	[169]
	Colorectal cancer	High	Oncogenic	PRMT6 cooperates with PRMT5 to epigenetically silence *CDKN2B* and CCNG1 through H3R2me2a	[81]
	Gastric cancer	High	Oncogenic	H3R2me2a suppresses tumor suppressor genes (*PCDH7*, *SCD*, and *IGFBP5*)	[79]
	Endometrial cancer	High	Oncogenic	PRMT6 promotes endometrial cancer via Akt/mTOR signaling	[296]
	Lung cancer	High	Oncogenic	PRMT6 interacts with ILF2 to drive alternative activation of tumor-associated macrophages	[220]
				Methylation of 6PGD (R324) and ENO1 (R9, R372) promotes glucose metabolism	[201]
	Glioblastoma	High	Oncogenic	PRMT6 attenuates the protein stability of CDKN1B by promoting its ubiquitinated degradation	[82]
				RCC1 methylation (R214) promotes chromatin association and RAN activation	[137]
	Melanoma	-	Tumor-suppressive	PRMT6 suppresses melanoma progression by depositing H3R2me2a at *ALDH1A1* promoter	[80]
	HCC	Low	Tumor-suppressive	CRAF methylation (R100) restrains RAS–MEK/ERK signaling, suppressing HCC progression	[124]
PRMT7					
	Breast cancer	High	Oncogenic	METTL3/IGF2BP1-driven m^6^A methylation enhances PRMT7 expression, activating Wnt signaling	[106]
				R531 automethylation promotes H4R3me2s and represses E-cadherin	[297]
				PRMT7 represses E-cadherin through H4R3me2s-mediated epigenetic remodeling	[160]
	Gastric cancer	Low	Tumor-suppressive	PRMT7 methylates PTEN and inhibits the PI3K/Akt pathway, suppressing gastric cancer progression	[122]
	Lung (NSCLC)	High	Oncogenic	PRMT7 promotes NSCLC metastasis through interaction with HSPA5 and EEF2	[298]
	Renal cell carcinoma	High	Oncogenic	PRMT7 methylates β-catenin and inhibiting the ubiquitin-mediated degradation of β-catenin	[118]
PRMT9					
	HCC	High	Oncogenic	PRMT9 promotes HCC invasion and metastasis by activating the PI3K/Akt/GSK-3β/Snail pathway	[123]
	AML	High	Oncogenic	PRMT9 drives AML progression by promoting leukemia cell survival and immune evasion	[217]

*AML* acute myeloid leukemia; acute megakaryoblastic leukemia, *ccRCC* clear cell renal cell carcinoma, *DLBCL* diffuse large B-cell lymphoma; *EBV* Epstein-Barr virus, *ESCC* esophageal squamous cell carcinoma, *HCC* hepatocarcinoma (hepatocellular carcinoma), *MTAP* methylthioadenosine phosphorylase, *NPC* nasopharyngeal carcinoma, *NSCLC* non-small cell lung carcinoma, *RCC* renal cell carcinoma, *SCLC,* small cell lung carcinoma

### Epigenetic and transcriptional regulation

**Fig. 4:**
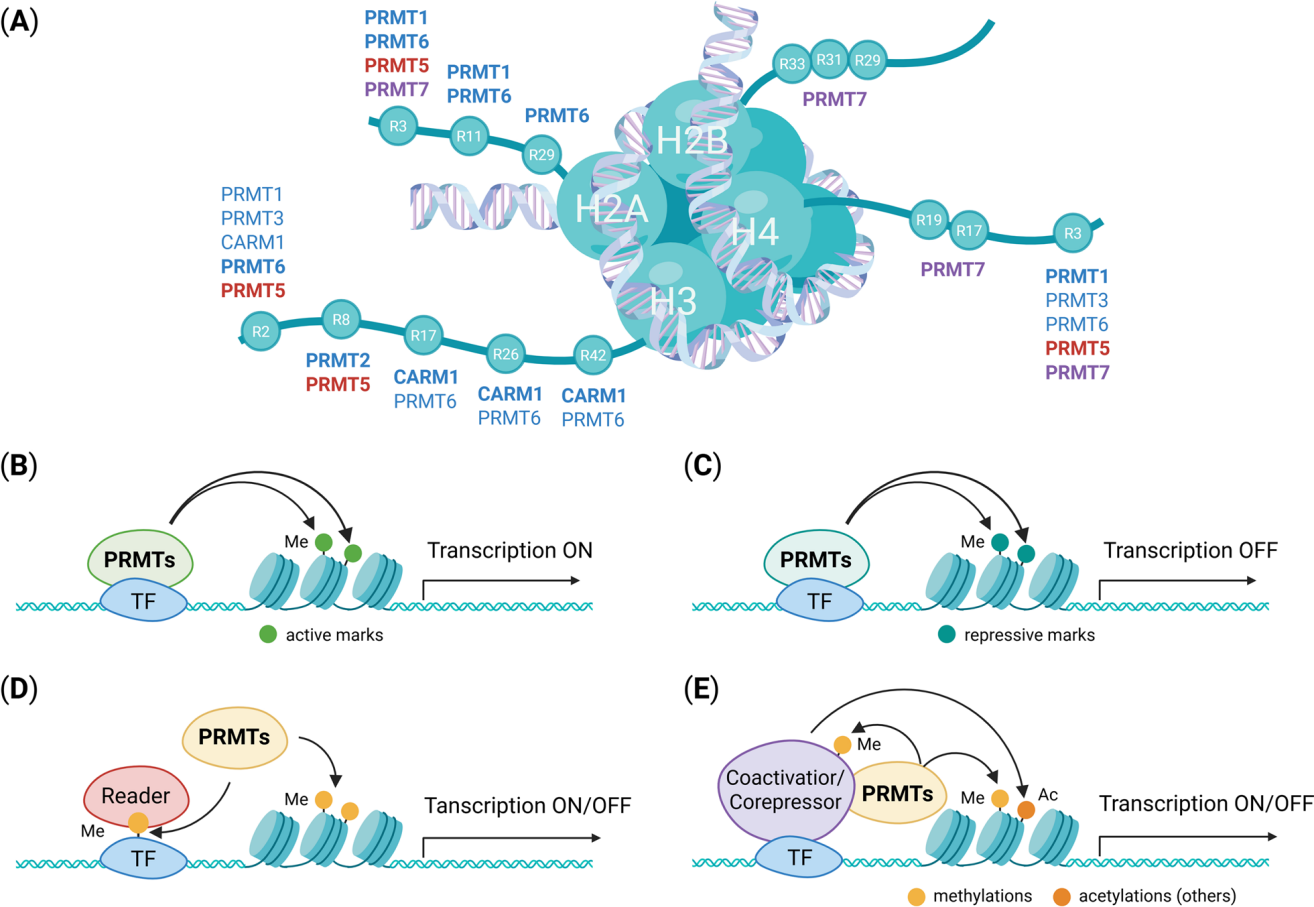
Histone arginine methylation and epigenetic regulation by PRMTs. **A** Schematic representation of arginine (R) residues on histone tails (H2A, H2B, H3, and H4) methylated by distinct PRMT family members. PRMT enzymes are color-coded as follows: Type I (blue), Type II (red), and Type III (purple). The indicated R residues represent established methylation sites targeted by specific PRMTs. **B** PRMTs deposit transcription-activating arginine methylation marks on histones, facilitating chromatin relaxation and gene activation. **C** PRMTs also generate transcription-repressive arginine methylation marks, promoting chromatin compaction and transcriptional silencing. **D** PRMTs methylate transcription factors (TFs), modulating their stability, localization, and DNA-binding activity to regulate gene expression. **E** PRMTs methylate transcriptional coactivators or corepressors and cooperate with other epigenetic modifications, such as histone acetylation, to fine-tune transcriptional outcomes. Green circles indicate transcriptionally active marks, whereas teal circles represent repressive marks. Yellow circles (Me) denote methylation events, and orange circles (Ac) represent acetylation

Epigenetic dysregulation is the central mechanism by which PRMTs drive oncogenesis. Several PRMT family members, particularly PRMT1, CARM1, and PRMT5, modulate chromatin architecture through site-specific methylation of histone arginine residues (Fig. 4). PRMT1-mediated asymmetric dimethylation of H4R3 (H4R3me2a) is associated with transcriptional activation through the recruitment of chromatin remodelers and histone acetyltransferases [62]. CARM1 catalyzes the asymmetric dimethylation of H3R17 (H3R17me2a) and H3R26 (H3R26me2a) [63]. At estrogen receptor (ER) target genes, CBP/p300 is typically recruited first to acetylate histones, thereby facilitating the subsequent recruitment of CARM1 and the establishment of H3R17me2a. This cooperative assembly of CBP/p300 and CARM1 enhances ER-driven transcription and oncogene expression in breast cancer [64, 65]. Conversely, PRMT5 catalyzes the symmetric dimethylation of H4R3 (H4R3me2s) and H3R8 (H3R8me2s), generating repressive chromatin environments that silence tumor-suppressor genes [66]. Besides histones, PRMTs modify numerous transcriptional regulators, including p53, NF-κB, E2F1, and MED12, which fine-tune transcriptional outputs that favor cell survival, proliferation, and malignant plasticity [5]. Collectively, aberrant histone and non-histone methylation by PRMTs remodels the epigenetic landscape, reinforcing transcriptional addiction and oncogenic signaling in cancer cells.

#### Histone modification

Histone arginine methylation is a fundamental step in epigenetic regulation that governs transcriptional programs. Asymmetric dimethylation by type I PRMTs favors transcriptional activation, whereas symmetric dimethylation by type II enzymes establishes a repressive chromatin state. The biological outcome depends on the specific modified residues: H4R3me2a, H3R8me2a, H3R17me2a, and H3R2me2s are activation marks, H4R3me2s and H3R8me2s correlate with repression, and H3R2me2a function bidirectionally [67].

PRMT1-mediated histone methylation recruits reader proteins and chromatin-remodeling complexes, which facilitate gene activation [68]. In colorectal cancer (CRC), PRMT1 enhances H4R3me2a deposition at the promoters of genes involved in growth and survival, in part through the recruitment of SMARCA4, the ATPase subunit of the SWI/SNF chromatin remodeling complex. This recruitment facilitates the transcriptional activation of EGFR and TNS4, thereby promoting tumor cell proliferation and migration [69]. In breast cancer, PRMT1 similarly facilitates *ZEB1* promoter methylation, inducing epithelial-mesenchymal transition (EMT) and cancer stem cell traits [70]. PRMT2-mediated H3R8me2a deposition also contributes to transcriptional activation. In glioblastoma (GBM), PRMT2 increases the expression of oncogenic clusters [71], and in renal carcinoma, PRMT2-dependent enrichment of H3R8me2a at the *WNT5A* promoter enhances Wnt signaling and tumor proliferation [72, 73]. CARM1-mediated H3R17me2a deposition likewise contributes to transcriptional activation. In various cancers, elevated H3R17me2a levels promote the expression of oncogenes and proliferation-related genes, thereby supporting tumor growth and progression [74, 75].

PRMT5 catalyzes the symmetric dimethylation of multiple residues, including H3R2, H3R8, and H4R3, which typically generates repressive chromatin states. Although PRMT5 exerts transcriptional repression through symmetric dimethylation, its effects vary depending on the chromatin context and its interacting partners. For example, PRMT5 represses *miR-29b* transcription via H4R3me2s in acute myeloid leukemia (AML) [76] and silences *CDKN2B* expression via H4R3me2s in CRC [77]. Conversely, in breast cancer stem cells, PRMT5 promotes *FOXP1* transcription through H3R2me2s [78], highlighting its dual role in epigenetic regulation of tumors.

Similar to PRMT5, PRMT6 mediates context-specific transcriptional regulation through H3R2me2a, a modification that modulates gene expression via crosstalk with H3K4me3. Depending on the chromatin landscape and target gene environment, PRMT6 can function as either a transcriptional repressor or activator, displaying tumor-suppressive or oncogenic effects. In cancer, PRMT6 enhances global H3R2me2a enrichment at tumor suppressor promoters such as *PCDH7* [79], and at oncogene promoters such as *ALDH1A1* [80]. Moreover, PRMT6 cooperates with PRMT5 to repress tumor suppressors such as *CDKN2B* and *CCNG1,* via the coordinated deposition of H3R2me2a, H4R3me2s, and H3R8me2s [81], establishing repressive chromatin environments that exert context-dependent effects on tumor progression. In contrast, PRMT6-mediated H3R2me2a also promotes *CDC20* transcription, resulting in the degradation of the cell cycle inhibitor *CDKN1B* and uncontrolled proliferation of GBM [82].

#### Non-histone modification

Beyond histone modifications, PRMTs profoundly influence transcription by methylating non-histone substrates, including transcription factors, nuclear receptors, coactivators, and chromatin remodelers. These modifications alter protein stability, DNA-binding affinity, and interactions with regulatory complexes, reprogramming oncogenic transcriptional networks.

*Transcription Factors*: Several PRMTs directly target transcription factors that act as master switches in oncogenic transcription. PRMT1 methylates multiple transcriptional regulators to modulate their stability and functions. In breast cancer cells, PRMT1 methylates C/EBPα at R35, R156, and R165 and disrupts its interaction with HDAC3, promoting cyclin D1 expression and increasing tumor cell proliferation [83]. PRMT1 also methylates ZEB1 to modulate EMT and cellular senescence [70]. In gastric cancer, PRMT1 recruits MLXIP to the *CTNNB1* promoter, activating Wnt/β-catenin signaling and promoting migration and metastasis [84]. Similarly, in AML models, CARM1 methylates RUNX1 at R223, enhancing its interaction with DPF2 and repressing *miR-223* transcription. Because miR-223 promotes myeloid differentiation, the knockdown of CARM1 reduces the leukemia burden [85]. In gastric cancer, PRMT5 interacts with c-Myc to transcriptionally repress tumor suppressor genes, including *CDKN1A*, *CDKN1C*, *CDKN2C*, *PTEN*, and *TP63*, promoting cell proliferation [86].

*Nuclear Receptors and Coactivators:* PRMT2 and CARM1 directly interact with ERα and function as transcriptional coactivators to enhance ERα-mediated gene expression [87]. Conversely, PRMT2 also suppresses ERα binding to the AP-1 site on the *CCND1* promoter, inhibiting its transcription in breast carcinoma cells [88]. PRMT5 activates androgen receptor (AR) transcription by interacting with Sp1 and recruiting the chromatin remodeler Brg1, promoting tumor progression in prostate cancer [89].

*Chromatin Remodelers and Epigenetic Enzymes:* PRMT1 methylates and stabilizes histone methyltransferase EZH2 at R342 by preventing CDK1- and AMPK-mediated phosphorylation and the TRAF6 ubiquitin–proteasome pathway. In addition, PRMT1-mediated EZH2 methylation enhances its binding to SUZ12 and PRC2 complex formation. This stabilization leads to the repression of *CDKN1A* and *CDKN2A* through H3K27me3 enrichment in their promoters, ultimately promoting EMT, invasion, and metastasis [90, 91]. CARM1 targets multiple components of the transcriptional machinery to promote oncogenic programs. It methylates the SWI/SNF complex subunit BAF155 at R1064, enhancing chromatin remodeling at oncogenic loci, including c-Myc target genes, driving cell migration and metastasis [92]. Methylated BAF155 also cooperates with BRD4 to activate oncogenic transcription while concurrently repressing ISG expression and reducing T-cell infiltration in metastatic tumors [93]. In addition, CARM1 methylates MED12 at R1862 and R1912, conferring resistance to chemotherapeutic agents, such as 5-FU and doxorubicin, by repressing *CDKN1A* transcription [94].

Together, these mechanisms underscore how PRMTs bridge transcriptional and epigenetic systems through non-histone substrate methylation, integrating multiple oncogenic signaling pathways into a coordinated transcriptional program.

### mRNA processing and translation regulation

PRMTs exert pivotal control over RNA metabolism by linking chromatin cues to post-transcriptional gene regulation. They fine-tune mRNA maturation, stability, and translation efficiency by methylating splicing factors, RNA-binding proteins, and translational machinery. Dysregulated arginine methylation in cancer cells disrupts these processes, fostering transcriptomic plasticity and oncogenic adaptation.

#### Regulation of splicing

PRMT5 is a critical regulator of pre-mRNA splicing. It methylates core spliceosomal components, including Sm proteins, enhancing their association with SMN [95]. In neural stem cells, the loss of PRMT5 disrupts the splicing of *MDM4*, reducing full-length transcript levels and generating a truncated isoform subject to nonsense-mediated decay (NMD). This destabilized isoform fails to properly inhibit p53, leading to defective cell cycle control [96]. In melanoma, loss of PRMT5 promotes exon skipping of *MDM4* to generate the MDM4-S isoform, restores p53 function, and sensitizes cells to CDK4/6 inhibitors [97]. Moreover, several splicing and RNA-processing factors, including Lsm4 and hnRNPH1, undergo PRMT5-dependent SDMA [96–98]. In GBM stem cells, PRMT5 inhibition causes widespread splicing defects, particularly in genes controlling the cell cycle and proliferation, suppressing tumor growth in vitro and in vivo [99].

Besides spliceosome assembly, PRMT-mediated methylation dynamically modulates alternative splicing in cancer. In breast cancer, PRMT1 methylates SRSF1 and enhances its RNA-binding activity, promoting oncogenic exon inclusion. Both PRMT1 and methylated SRSF1 are upregulated in tumors, and their inhibition attenuates aberrant splicing and tumor growth [100]. Similarly, in AML, PRMT5 methylates SRSF1 at R93, R97, and R109, stabilizing RNA–protein interactions and promoting the efficient splicing of proliferation-related transcripts. Loss of PRMT5 disrupts these networks, causing extensive alternative splicing and cell death [101]. PRMT5 also methylates ZNF326 at R175, which is essential for RNA polymerase II transcription of A-T-rich genes. Loss of PRMT5 induces A-T-rich exon inclusion in *ST3GAL5*, *FOXM1*, and *AP4*, generating aberrant transcripts that are degraded by NMD. These defects impair breast cancer cell proliferation and migration [102]. Collectively, PRMT-dependent regulation of alternative splicing ensures precise RNA maturation, whereas its dysregulation contributes to malignant transformation and therapeutic resistance in various cancers.

#### mRNA modification and metabolism

The N^6^-methyladenosine (m^6^A) modification is a major determinant of mRNA metabolism, influencing transcript splicing, export, translation, and decay [103]. The METTL3–METTL14–WTAP complex functions as the principal m^6^A methyltransferase. PRMT1 is linked to the m^6^A machinery by methylating METTL14 at R442 and R445. This modification promotes the association of METTL14 with RNA polymerase II, enhancing m^6^A deposition on transcripts involved in DNA interstrand crosslink repair pathways. PRMT1 maintains genomic stability under genotoxic stress through METTL14 methylation; consequently, its inhibition sensitizes cancer cells to chemotherapy [104, 105]. The m^6^A machinery acts upstream of PRMT7. The METTL3/IGF2BP1 axis enhances m^6^A modification of PRMT7 mRNA, stabilizing its expression and activating Wnt/β-catenin signaling to promote tumor progression [106].

Besides m^6^A regulation, PRMT1 exerts broad control over RNA metabolism. Multiomics analyses have identified PRMT1 as a central regulator that integrates RNA processing and DNA damage response (DDR) networks. In pancreatic ductal adenocarcinoma (PDAC), PRMT1 interacts with RNA-binding proteins such as hnRNPs, coordinating RNA splicing and genome maintenance [107]. Similar observations in clear cell renal cell carcinoma (ccRCC) revealed that PRMT1 loss leads to R-loop accumulation and double-stranded DNA breaks, ultimately triggering growth arrest [108]. These findings suggest that PRMT1 is a key regulator of RNA metabolism, genomic integrity, and cancer cell survival.

#### Translational regulation

PRMTs are crucial regulators of translational homeostasis and influence both global and selective protein synthesis. In p53/Rb-deficient osteosarcoma, PRMT1 regulates global translation by methylating core components of the translation initiation complex, including eIF4G1, eIF4A, and eIF4E. This suggests that PRMT1 is an oncogenic driver that safeguards translation under stress conditions and highlights it as a potential therapeutic target [109]. In addition, PRMT5 regulates internal ribosome entry site (IRES)-dependent translation by methylating hnRNP A1 at R218 and R225. This methylation enhances the affinity of hnRNP A1 for IRES-containing mRNAs, such as *CCND1* and *MYC*, promoting translation initiation. Mutations in these residues or PRMT5 inhibition disrupt hnRNP A1–IRES binding and selectively impair IRES-dependent translation. Through this mechanism, PRMT5 supports the synthesis of proteins encoded by *CCND1*, *MYC*, *HIF1A,* and *ESR1*, promoting tumor proliferation and survival [110].

### Signal transduction and cell cycle regulation

PRMTs have emerged as central integrators of oncogenic signaling and cell cycle control. Extracellular cues are linked to transcriptional and checkpoint responses through methylation of both histone and non-histone substrates. At the signaling level, PRMTs modify receptors, kinases, and transcription factors across major oncogenic pathways, including EGFR, TGF-β, Wnt, PI3K–Akt, MAPK, and NF-κB, fine-tuning the amplitude and duration of signal transduction [7]. At the downstream effector level, PRMT-dependent methylation of key cell cycle regulators, including p21, cyclin E1, and CDK1, converts upstream inputs into cellular decisions of proliferation or arrest [5]. Collectively, PRMTs function as methylation-based rheostats that integrate growth factor signaling with sustained tumor cell proliferation and metastatic progression.

#### Signal transduction

*EGFR pathway:* PRMT1 methylates EGFR at R198 and R200 within the extracellular domain, enhancing EGF binding, receptor dimerization, and downstream signaling in CRC cells [111]. PRMT5 also promotes EGF-induced EGFR trans-autophosphorylation by methylating EGFR at R1199 (corresponding to R1175 in mature EGFR) [112]. In pancreatic cancer cells, PRMT5 is upregulated and enhances EGFR phosphorylation and downstream Akt and GSK3β activation, leading to increased β-catenin expression. This PRMT5-dependent signaling cascade promotes the expression of EMT-related genes, such as vimentin and collagen I [113].

*TGF-β/SMAD pathway:* PRMT1 promotes TGF-β–driven EMT by methylating SMAD7 at R57 and R67, enhancing the transcription of EMT- and stemness-associated genes in mammary epithelial cells [114]. In CRC cells, PRMT5 also reinforces TGF-β signaling through SMAD4 methylation at R361, facilitating SMAD complex formation and nuclear translocation to induce EMT and metastasis. Clinically, elevated PRMT5 expression and increased SMAD4 R361 methylation correlate with poor patient prognosis [115].

*Wnt/*β*-catenin pathway:* Aberrant activation of Wnt/β-catenin signaling is a hallmark of CRC. CARM1, which is frequently overexpressed in colon cancer, interacts with β-catenin to enhance β-catenin–driven transcription. β-catenin recruits CARM1 to LEF/TCF-bound promoters, where CARM1 deposits H3R17me2a, creating an active chromatin state that promotes target gene expression and cell proliferation [116]. In hepatocellular carcinoma (HCC), the MTDH–PRMT5 complex augments Wnt/β-catenin signaling. Overexpressed MTDH preferentially binds to PRMT5, releasing β-catenin for nuclear translocation and activating downstream oncogenic programs [117]. In ccRCC, PRMT7 is upregulated and methylates β-catenin, protecting it from ubiquitin-mediated degradation and amplifying the β-catenin/c-Myc axis to drive cell proliferation [118].

*PI3K/Akt/mTOR pathway:* PRMT5 directly enhances PI3K/Akt signaling through multiple methylation events in Akt. PRMT5-mediated methylation of Akt1 at R391, along with phosphatidylinositol (3,4,5)-trisphosphate, weakens intramolecular PH–KD binding, facilitating membrane translocation and subsequent activation of PDK1 and mTORC2 [119]. PRMT5 also methylates Akt1 at R15, enabling its full activation via phosphorylation at T308 and S473. PRMT5 inhibition abolishes these events, impairs Akt activation, and suppresses EMT transcription factors, such as ZEB1, Snail, and Twist1, reducing neuroblastoma growth and metastasis [120]. In GBM, PRMT5 indirectly sustains PI3K/Akt signaling by repressing *PTEN*, which promotes proliferation through the suppression of p27 and activation of E2F targets. PRMT5 depletion reversed these effects and induced G1/S arrest and cellular senescence. In contrast, PRMT6 counteracts the PI3K/Akt pathway by methylating PTEN at R159 [121]. Other PRMT family members modulate this axis in a context-dependent manner. In gastric cancer, PRMT7 promotes PTEN methylation and activates PI3K/Akt signaling [122]. In HCC, PRMT9 activates the PI3K/Akt/GSK3β/Snail cascade, promoting EMT and metastasis [123].

*RAS/RAF/MEK/ERK pathway:* PRMT6 is frequently downregulated in HCC, and its expression is inversely correlated with aggressive cancer features in patients with HCC. PRMT6 silencing promoted tumorigenesis, metastasis, and therapeutic resistance in HCC cell lines and patient-derived organoids. PRMT6 methylates CRAF at R100, reducing its RAS-binding potential and inhibiting downstream MEK/ERK signaling. Consequently, PRMT6 deficiency upregulates stemness-related genes, such as *CD133*, *SOX2*, and *NANOG* [124].

*NF-κB pathway:* PRMT5 amplifies NF-κB signaling by methylating YBX1 at R205 and p65 at R30. These modifications strengthen YBX1–p65 interactions and enhance p65 DNA binding, driving the transcription of YBX1-dependent NF-κB target genes and promoting oncogenic and proinflammatory responses [125]. In bladder cancer, PRMT5 facilitates NF-κB recruitment to the promoters of anti-apoptotic genes, such as *BCLXL* and *BIRC2*, suppressing apoptosis [126].

#### Cell cycle regulation

*G1/S Transition and Checkpoint Control:* CARM1 contributes to the G1/S transition by regulating ERα-mediated transcriptional activation. Upon estrogen stimulation, CARM1 associates with ERα and the coactivator AIB1 to promote H3R17me2a at the *E2F1* promoter, thereby enhancing E2F1 transcription and facilitating cell cycle progression [75]. In a growth stimulation context, CARM1 is recruited to the *CCNE1* promoter in an E2F-dependent manner, together with the p160 coactivator ACTR. This recruitment is accompanied by dynamic changes in H3R17 and H3R26 methylation and contributes to *CCNE1* activation and S-phase entry [127]. Furthermore, during the G1/S transition, CARM1-mediated methylation of Rb at R775, R787, and R798 enhances CDK-dependent phosphorylation and disrupts its association with E2F1, activating E2F1 target genes and driving G1/S progression [128]. Recent evidence further demonstrates that CARM1 hypermethylates components of the NuRD chromatin remodeling complex, including GATAD2A/B, thereby enhancing the expression of cell cycle–related genes and promoting breast cancer development [129].

PRMT5 is essential for cell proliferation because it sustains the G1/S transition. It directly methylates E2F1 at R111 and R113, reducing protein stability. Under DNA damage stress, methylation decreases, leading to E2F1 accumulation and induction of apoptosis [130]. Consistently, PRMT5 overexpression promotes tumor cell growth in epithelial ovarian cancer, whereas its inhibition triggers apoptosis via E2F1 upregulation [131]. In addition, PRMT5 depletion suppresses p53 protein synthesis by downregulating the translation factor eIF4E, resulting in impaired induction of p53 target genes, such as *MDM2* and *CDKN1A*, upon DNA damage. Together, these findings establish PRMT5 as a key pro-survival regulator that integrates methylation–dependent control of E2F1 stability and p53 translation to sustain cell cycle progression [132].

*G2/M Transition and Mitotic Control:* PRMTs orchestrate multiple steps in mitotic regulation through histone and non-histone methylation. Inhibition of PRMT1 activates the p53/p21 signaling pathway, suppressing cyclin B and CDK1, which leads to G2/M arrest and accumulation of mitotic cells [133]. CARM1 plays a multifaceted role in mitosis. CARM1-mediated methylation of PI3KC2α at R175 enhances its interaction with tubulin, stabilizes microtubules, and promotes proper spindle formation [61, 134, 135]. Besides its methyltransferase activity, CARM1 functions as a scaffold that regulates CDK1 stability [42]. During interphase, CARM1 acts as an adaptor for Cullin-1-mediated CDK1 degradation, restricting nuclear CDK1 levels. In late G2, the CDK1–cyclin B1 complex translocates to the nucleus and phosphorylates CARM1, inactivating its enzymatic function and inducing its cytoplasmic translocation. Loss of nuclear CARM1 stabilizes the nuclear CDK1–cyclin B1 complex, facilitating mitotic entry.

Additional layers of mitotic regulation are provided through histone arginine methylation. Upon mitotic entry, CARM1 is phosphorylated by CDK1 and PKC, leading to enzymatic inactivation and a decrease in H3R17me2a levels [42, 59]. Concurrently, PRMT6 deposits the H3R2me2a mark [42, 136]. These coordinated chromatin modifications are essential for the recruitment of the chromosomal passenger complex (CPC), facilitating Aurora B binding to chromatin and promoting H3S10 phosphorylation, a key step in chromosome condensation. Loss of H3R2me2a impairs CPC localization to chromosomal arms and disrupts mitotic progression [136]. In GBM, CK2α phosphorylates and stabilizes PRMT6, enhancing the PRMT6-dependent methylation of RCC1 at R214. This modification promotes chromatin association and Ran GTPase activation, facilitating mitotic progression and nucleocytoplasmic transport during the interphase [137].

**Fig. 5:**
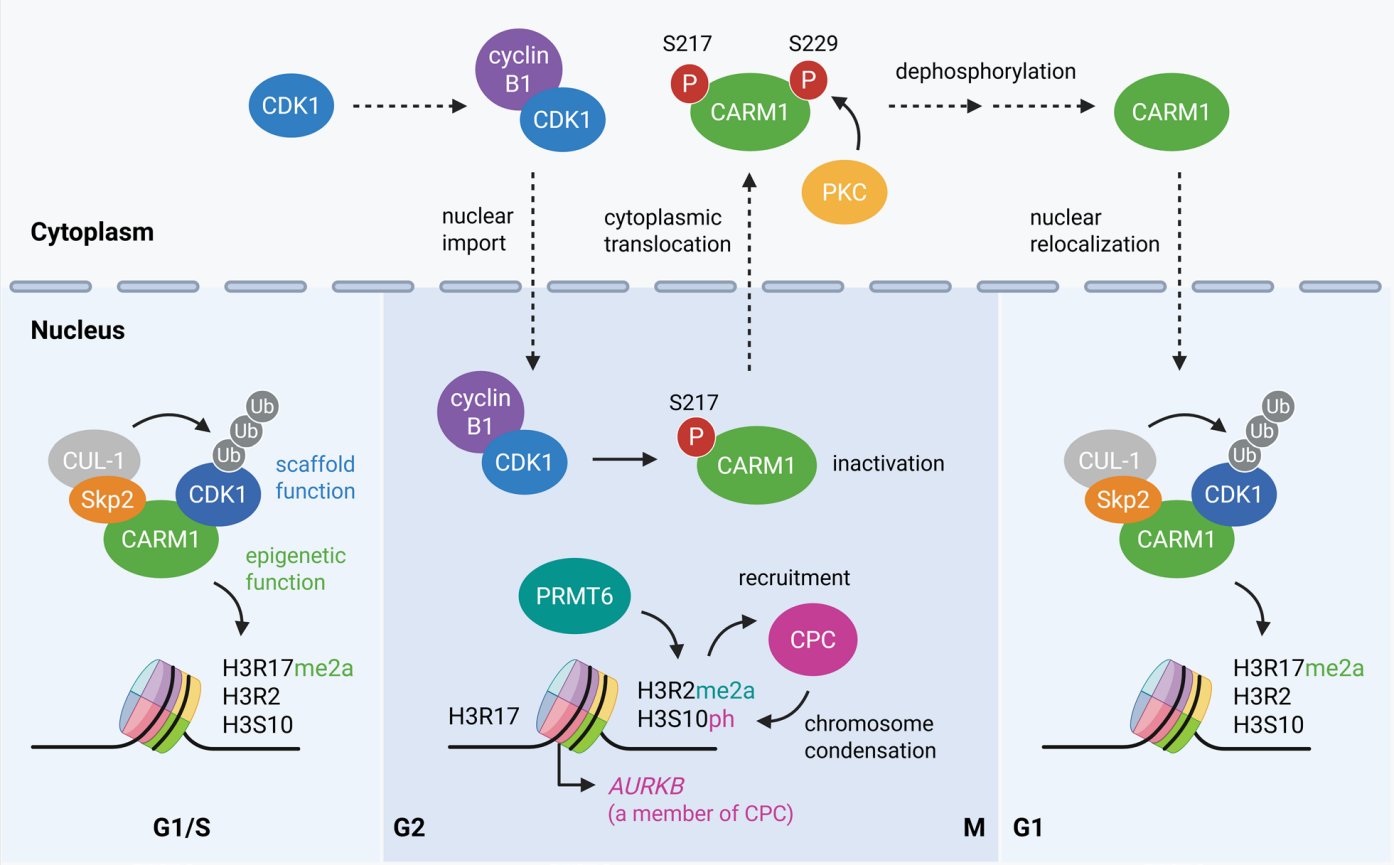
Temporal regulation of mitosis by PRMTs. During interphase (G1/S), CARM1 (green) localizes to the nucleus, where it acts as a scaffold to facilitate Cullin-1 (CUL1)–Skp2–mediated ubiquitination of CDK1 (blue), thereby maintaining low nuclear CDK1 levels while exerting epigenetic functions such as H3R17me2a deposition. As cells enter late G2, the CDK1–cyclin B1 complex (blue/purple) accumulates and translocates into the nucleus. CDK1 phosphorylates CARM1 at S217, suppressing its methyltransferase activity and promoting cytoplasmic relocalization. Loss of nuclear CARM1 disrupts CDK1 ubiquitination, resulting in CDK1 stabilization and sustained nuclear retention of the CDK1–cyclin B1 complex to drive mitotic entry. During mitosis (M phase), PRMT6 (teal) catalyzes H3R2me2a, recruiting the chromosomal passenger complex (CPC) to chromosome arms. This enhances Aurora B (*AURKB*)-dependent H3S10 phosphorylation and promotes chromosome condensation. Upon mitotic exit, CARM1 re-enters the nucleus, restores CDK1 ubiquitination, and resets the cell-cycle regulatory circuit. Color scheme: CDK1 (blue), cyclin B1 (purple), CARM1 (green), PRMT6 (teal), and CPC components (magenta). Yellow circles (Me) indicate methylation, red circles (P) indicate phosphorylation, and gray (Ub) symbols denote ubiquitination

Collectively, these findings demonstrate that PRMTs coordinate mitotic progression through diverse mechanisms, including epigenetic regulation, scaffold function, and kinase-driven signaling (Fig. 5). In particular, the dual roles of CARM1—as a methyltransferase regulating spindle formation and chromosome condensation, and as an adaptor modulating CDK1 homeostasis—underscore its central role in maintaining mitotic integrity.

### DNA damage repair and genome stability

PRMTs are pivotal regulators of DDR, modulating the recruitment, activity, and stability of repair factors through both histone and non-histone methylation. By targeting core DNA repair proteins and chromatin components, PRMTs orchestrate multiple DNA repair pathways, including homologous recombination (HR), non-homologous end joining (NHEJ), base/nucleotide excision repair (BER/NER), and replication-associated checkpoint signaling [138].

#### DNA double-strand break repair

**Fig. 6:**
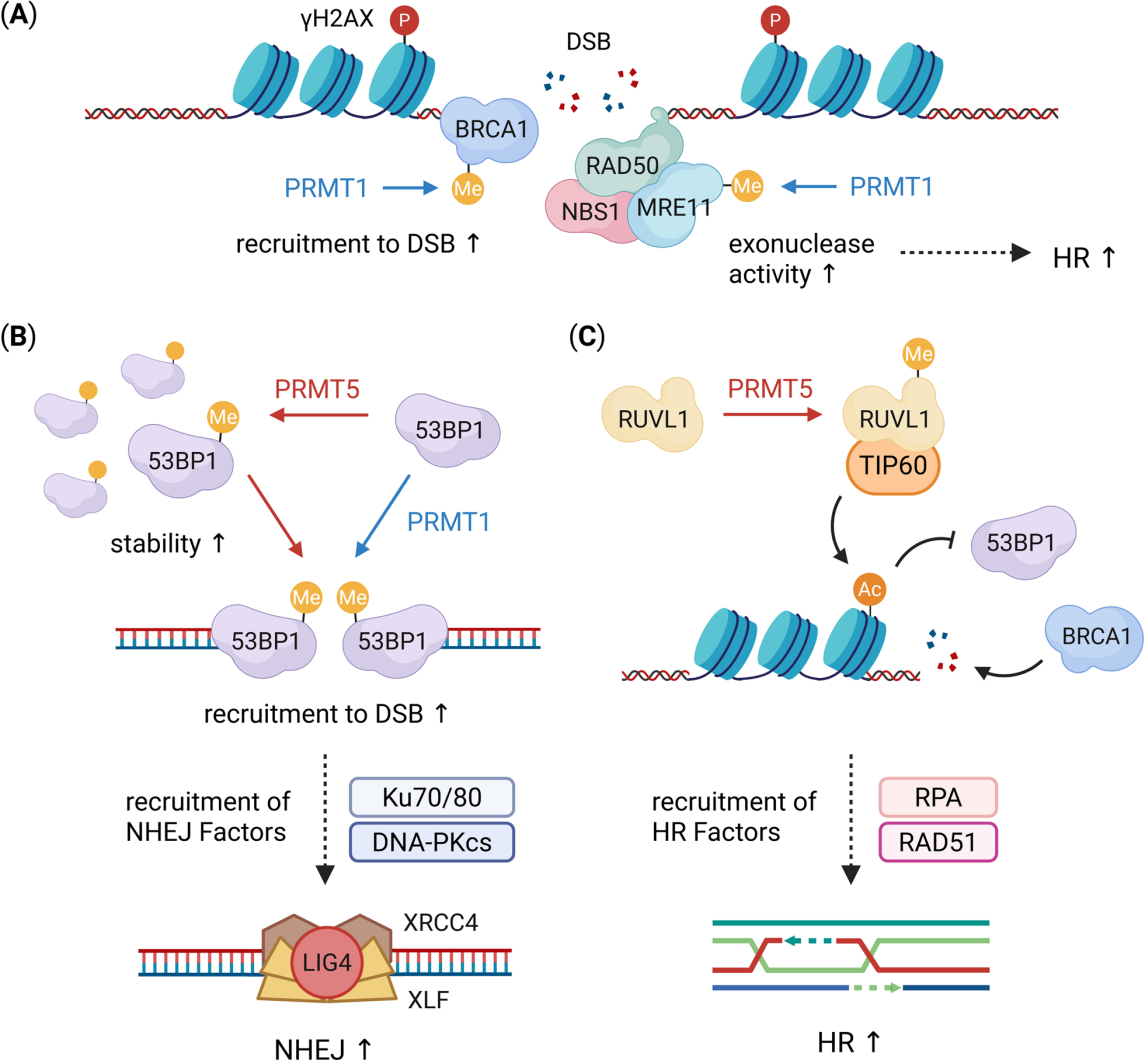
Regulation of DDR by PRMTs. **A** Following DNA double-strand breaks (DSBs), PRMT1 (blue) methylates MRE11 within the MRN complex (MRE11–RAD50–NBS1), enhancing its exonuclease activity and promoting homologous recombination (HR). PRMT1 also methylates BRCA1, contributing to HR regulation. **B** In the non-homologous end joining (NHEJ) pathway, 53BP1 is methylated by both PRMT1 (blue) and PRMT5 (red). PRMT5-mediated methylation increases 53BP1 stability, whereas PRMT1-mediated methylation enhances its recruitment to DSB sites. Elevated 53BP1 recruitment facilitates the assembly of NHEJ factors, including Ku70/80, DNA-PKcs, XRCC4, XLF, and LIG4, thereby promoting NHEJ. **C** In HR regulation, PRMT5 (red) methylates RUVBL1, facilitating TIP60-dependent histone acetylation (Ac). This modification antagonizes 53BP1 recruitment and promotes the loading of HR factors, including RPA and RAD51, thereby enhancing HR-mediated repair. Yellow circles (Me) indicate methylation events, orange circles (Ac) represent acetylation, and red circles (P) denote phosphorylation

Double-strand DNA breaks (DSBs) are among the most lethal DNA lesions and are primarily repaired by HR and NHEJ. PRMTs have emerged as critical modulators of these pathways, as they orchestrate the assembly and function of repair factors (Fig. 6). PRMT1-mediated BRCA1 methylation determines the binding preference for Sp1 or STAT1 [139], promoting chromatin recruitment. In breast cancer, loss of PRMT1 mislocalizes BRCA1 to the cytoplasm, resulting in defective DNA repair and increased radiosensitivity [140]. PRMT1 methylates both MRE11 and 53BP1, promoting their recruitment to DSBs [141–143]. These methylation events are facilitated by GFL1, which serves as an adaptor that enables PRMT1 to interact with MRE11 and 53BP1 [144]. In addition, DNA-PK-dependent PRMT1 phosphorylation drives PRMT1 accumulation in chromatin upon cisplatin exposure, inducing the expression of senescence-associated secretory phenotype genes through sustained H4R3me2a deposition [145].

PRMT5 orchestrates the cellular choice between NHEJ and HR through the multilayered regulation of DNA repair factors. At the protein stability level, PRMT5 methylates and stabilizes 53BP1, promoting NHEJ, which is counteracted by Src-mediated phosphorylation, inhibiting PRMT5 and diminishing 53BP1 accumulation [50]. PRMT5 also modulates the functional engagement of repair proteins in DSBs. Methylation of RUVBL1 at R205 facilitates TIP60/KAT5-dependent chromatin acetylation and displaces 53BP1 from DSBs, suppressing NHEJ [146]. In parallel, PRMT5-mediated METTL3 methylation at R36 enhances RAD51 recruitment to DSBs, promoting HR [147]. Besides its direct effects on repair factor stability and recruitment, PRMT5 exerts a broader influence on repair pathways by modulating mRNA splicing. Loss of PRMT5 results in the aberrant splicing of key chromatin-modifying enzymes, such as TIP60/KAT5 and KMT5C/SUV4-20H2, leading to reduced TIP60α expression and impaired chromatin acetylation, ultimately compromising HR efficiency [148].

CARM1 contributes to BRCA1 regulation by methylating CBP/p300 at R754, a modification that is recognized by the BRCT domain of BRCA1. This interaction facilitates the recruitment of BRCA1 to the p53-binding region of the *CDKN1A* promoter [149]. Besides this transcription-coupled mechanism, CARM1 is rapidly recruited to DSBs via its interaction with PARP1, where it contributes to efficient DSB repair [150].

#### Base excision repair

PRMT6 enhances BER efficiency by methylating DNA polymerase β at R83 and R152. These modifications increase DNA-binding affinity and processivity, promoting more efficient repair synthesis and conferring resistance to alkylation-induced DNA damage [151]. PRMT1 methylates DNA polymerase β at R137, disrupting its interaction with PCNA. This methylation likely modulates the handoff between BER and replication, preventing the inappropriate engagement of DNA polymerase β at replication forks and ensuring pathway fidelity [152]. In NER, the structure-specific endonuclease XPF–ERCC1 is essential for incising damaged DNA strands, particularly during the removal of ultraviolet (UV)-induced pyrimidine dimers. CARM1 methylates XPF at multiple arginine residues, including R568, which is required for XPF protein stability, chromatin association, and efficient heterodimerization with ERCC1. Therefore, loss of CARM1 reduces XPF–ERCC1 levels and impairs its recruitment to UV-damaged chromatin, leading to impaired NER efficiency and heightened sensitivity to UV irradiation [153].

#### Damage sensing and checkpoint signaling

PRMT5 regulates genomic integrity through multiple mechanisms, including the control of γH2AX proteostasis, checkpoint signaling, and transcriptional regulation. PRMT5 balances γH2AX stability by modulating ubiquitination through the PRMT5–RNF168–SMURF2 axis: RNF168 stabilizes γH2AX, whereas SMURF2 promotes its degradation. Specifically, PRMT5 preserves γH2AX levels by maintaining RNF168 expression via H3R2me1 and H3R8me2s. In GBM, loss of MTAP disrupts this pathway, leading to impaired DNA damage signaling [154]. PRMT5-mediated RAD9 methylation at R172, R174, and R175 are also required for genotoxin-induced Chk1 phosphorylation. Methylated and phosphorylated RAD9 subsequently forms a 9–1-1 complex with RAD1 and Hus1, which is critical for cell cycle control and DNA repair [155]. In addition, PRMT5 methylates and stabilizes the transcription factor KLF4 at R374, R376, and R377, promoting cell survival by inducing *CDKN1A* and repressing *BAX*. Upon DNA damage, the loss of KLF4 methylation triggers its degradation, leading to cell cycle arrest [156].

### Tumor metastasis

PRMTs are key epigenetic regulators that drive cancer metastasis by orchestrating processes essential for tumor dissemination, survival, and colonization. Metastasis typically begins with EMT, during which epithelial cancer cells lose polarity and adhesion, while gaining motility and invasive potential. PRMTs promote EMT by repressing epithelial markers, such as E-cadherin (*CDH1*), and activating mesenchymal markers, including vimentin (*VIM*), either directly or indirectly through modulation of EMT-inducing transcription factors (EMT-TFs) [157, 158]. PRMT1 methylates and stabilizes EZH2 at R342, reinforcing H3K27me3-dependent repression of *CDH1* [91]. Similarly, CARM1 methylates and stabilizes LSD1 at R838, repressing *CDH1* and activating *VIM* transcription through H3K4me2 and H3K9me2 [159]. PRMT7-mediated H4R3me2s also inhibits *CDH1* expression by reducing H3K4me3, H3Ac, and H4Ac at the *CDH1* promoter during EMT induction [160]. In addition, PRMTs regulate key EMT-TFs. Snail and Slug form complexes with PRMT5 and LSD1 to repress *CDH1* and activate *VIM* transcription [161, 162]. ZEB2 cooperates with Twist1, PRMT5, and the NuRD complex to epigenetically silence *CDH1*, reinforcing the mesenchymal phenotype [163]. PRMT1-mediated methylation of Twist1 at R34 strengthens its repressor function, whereas PRMT1 enhances *ZEB1* expression via H4R3me2a deposition in its promoter [70, 164].

At the signaling level, PRMTs modulate key pathways that govern EMT and metastasis. In the TGF-β pathway, PRMT1 and CARM1 methylate SMAD6 and SMAD7, promoting their dissociation from receptors and enhancing SMAD-dependent transcription [114, 165, 166]. PRMT5 methylates SMAD4 at R361, facilitating its nuclear translocation and transcriptional activity [115]. PRMT5 also potentiates Wnt signaling by epigenetically silencing pathway antagonists, such as DKK1 and DKK3, leading to enhanced β-catenin–driven transcriptional programs [167, 168]. Furthermore, PRMT5-mediated Akt1 methylation at R15 and PRMT1/PRMT6-dependent STAT3 methylation activate downstream oncogenic signaling, promoting EMT and metastatic potential [120, 169]. Beyond these pathways, PRMTs modulate growth factor receptor signaling, including that of EGFR and FGFR3, to enhance migration, invasion, and EMT induction.

Parallel to EMT regulation, PRMTs promote cancer cell migration and invasion through cytoskeletal remodeling, adhesion turnover, and extracellular matrix degradation. PRMT1-driven methylation of NONO enhances CRC metastasis [170], and PRMT3 promotes breast cancer proliferation and metastasis through H4R3me2a-dependent endoplasmic reticulum stress signaling [171]. CARM1 activates genes that enhance cancer cell migration, invasion, and metastasis by methylating BAF155 [93]. PRMT5 promotes cancer metastasis by recruiting DNMT3A to the promoter regions of tumor suppressor genes [172]. Moreover, PRMT5 functions as a scaffold of the glucocorticoid receptor—independent of its enzymatic activity—by recruiting phospho-HP1γ and RNA polymerase II, enhancing glucocorticoid receptor-dependent gene transcription and promoting TNBC cell motility [173]. PRMT6 supports tumor growth and metastasis by modulating circadian gene expression [174]. PRMTs also enable cell survival during metastatic dissemination by conferring resistance to anoikis and apoptosis triggered by the loss of extracellular matrix attachment. PRMT1 methylates and stabilizes PGC-1α to promote anoikis resistance [175], and PRMT5 methylates MTHFD1 to increase NADPH production, facilitating metabolic adaptation under anchorage-independent conditions [176].

Collectively, PRMTs orchestrate a multifaceted pro-metastatic program that encompasses EMT induction, EMT-TF modulation, signaling network regulation, cytoskeletal remodeling, and metabolic adaptation. These diverse functions highlight PRMTs as pivotal drivers of tumor progression and as compelling therapeutic targets for preventing metastatic cancer dissemination.

### Metabolic reprogramming and stress adaptation

**Fig. 7:**
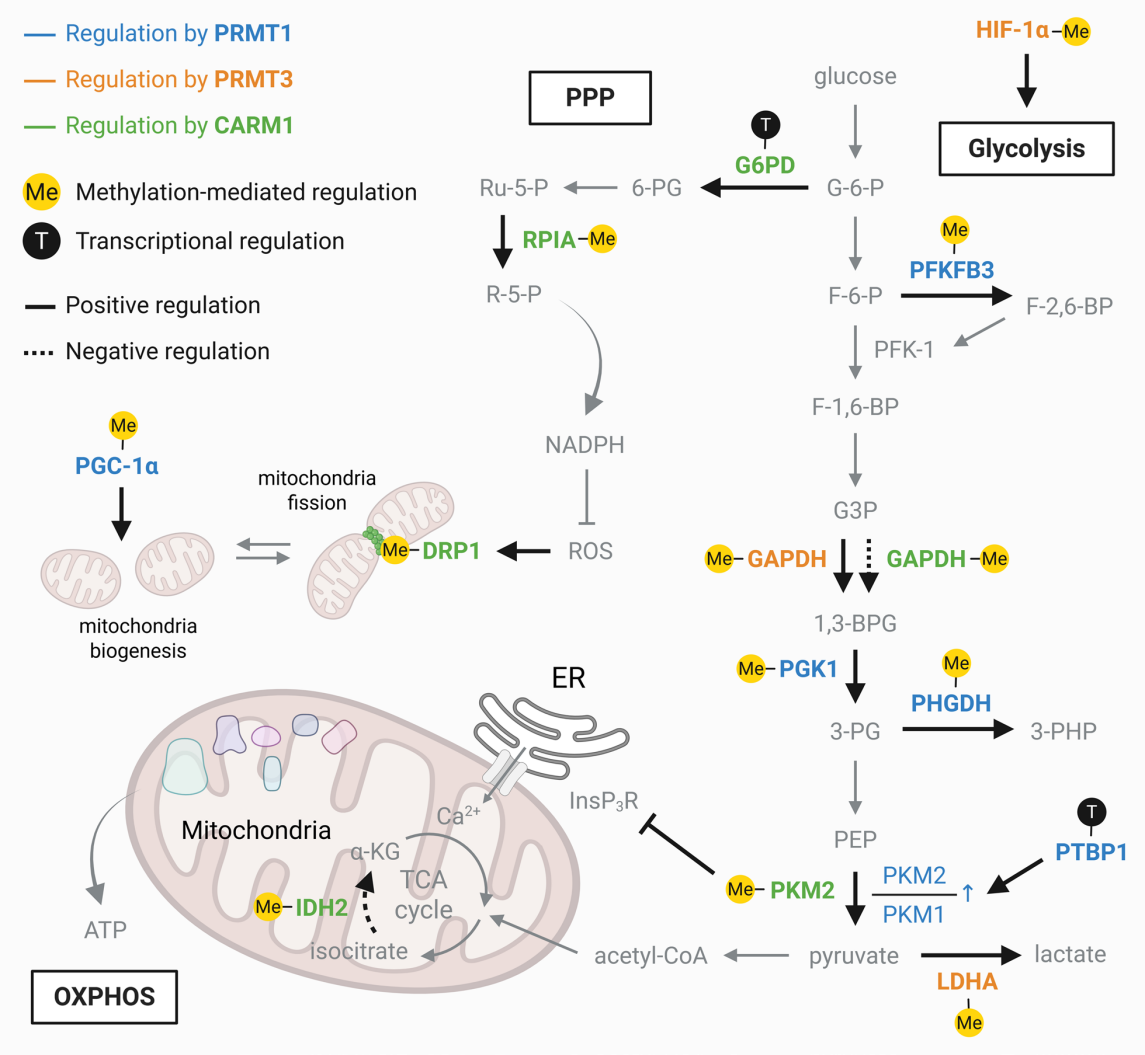
PRMT-driven metabolic reprogramming in cancer. PRMTs orchestrate metabolic reprogramming by coordinating glycolysis, the pentose phosphate pathway (PPP), serine biosynthesis, and mitochondrial function to support tumor growth. PRMT1 (blue), PRMT3 (orange), and CARM1 (green) regulate glycolytic flux through transcriptional control and direct methylation of key metabolic enzymes, including G6PD, RPIA, PFKFB3, GAPDH, PGK1, PHGDH, PKM2, PTBP1, LDHA, and IDH2. Yellow circles (Me) indicate methylation-mediated regulation, whereas black circles (T) denote transcriptional regulation. Solid lines represent positive regulation and dotted lines indicate negative regulation. These modifications enhance glucose utilization, lactate production, and promote the PKM2/PKM1 isoform switch. CARM1-mediated activation of G6PD and methylation of RPIA stimulate the PPP, elevate NADPH levels, and maintain redox homeostasis. CARM1-dependent methylation of DRP1 and IDH2, along with modulation of mitochondrial Ca^2^⁺ signaling, regulates mitochondrial dynamics, TCA cycle activity, and oxidative phosphorylation (OXPHOS). Collectively, PRMTs integrate cytoplasmic and mitochondrial metabolism to increase metabolic flexibility in cancer cells

Metabolic reprogramming is a hallmark of cancer that enables tumor cells to proliferate rapidly and persist in nutrient-limited conditions [177, 178]. Among the central regulators, PRMTs have emerged as pivotal orchestrators of glucose metabolism and the Warburg effect. However, their metabolic influence extends far beyond glycolysis, encompassing amino acid synthesis, redox balance, and lipid metabolism, collectively shaping multiple layers of metabolic control in cancer (Fig. 7).

#### Glycolysis, glucose utilization, and energy homeostasis

Several PRMTs promote aerobic glycolysis by regulating key glycolytic enzymes. PRMT1 drives glucose metabolism through multiple mechanisms. In non-small cell lung cancer (NSCLC), PRMT1-deposited H4R3me2a upregulates PTBP1, which enhances the glycolytic flux by increasing the PKM2/PKM1 ratio [179]. In CRC, PRMT1 methylates PGK1 at R205, promoting its phosphorylation and shifting energy production toward glycolysis [180]. In acute megakaryocytic leukemia, PRMT1 increases glucose consumption while suppressing CPT1A-dependent fatty acid oxidation, making cells metabolically reliant on glycolysis [181]. PRMT3 also enhances glycolytic flux through both direct enzymatic regulation and transcriptional control. At the enzymatic level, PRMT3 methylates LDHA at R112 in hepatocellular carcinoma, thereby increasing lactate production [182], and GAPDH at R248 in pancreatic cancer, promoting the assembly of its active tetrameric structure and enhancing catalytic activity [183]. In addition, PRMT3-mediated methylation of HIF-1α at R282 increases protein stability [184], thereby promoting the transcription of glycolytic genes, including *PGK1*, *PDK1*, *GAPDH*, *TPI1*, *LDHA,* and *PFKL* [185]. CARM1 functions as both a positive and negative regulator of glycolysis: it promotes glycolytic commitment by methylating PKM2 at R445, R447, and R455 [186], while suppressing glycolysis through methylation of GAPDH at R234 [187]. In breast cancer cells, CARM1-mediated methylation of PKM2 reduces the expression of inositol-1,4,5-trisphosphate receptors and limits Ca^2^⁺ transfer from the endoplasmic reticulum to mitochondria, thereby attenuating mitochondrial oxidative metabolism and promoting a shift toward aerobic glycolysis [186]. In liver cancer cells, CARM1-mediated methylation of GAPDH suppresses its catalytic activity and represses glycolysis in an AMPK-dependent manner [187]. Beyond cytosolic glycolysis, CARM1 also links glucose metabolism to mitochondrial energy homeostasis. CARM1 localizes to mitochondria and contributes to energy homeostasis by targeting key tricarboxylic acid (TCA) cycle enzymes [60]. Notably, methylation of IDH2 at R188 suppresses its catalytic activity while enhancing protein stability [60]. Consistent with this role, CARM1 inhibition has been reported to increase oxygen consumption rates and enhance oxidative phosphorylation [60, 188, 189], suggesting that CARM1 may restrain mitochondrial respiratory activity.

#### Amino acid and redox metabolism

PRMTs also redirect carbon flux toward amino acid synthesis and redox regulation. PRMT1 methylates PHGDH at R236, activating serine biosynthesis and alleviating oxidative stress in hepatocellular carcinoma [190]. Under proliferative conditions, PRMT1 methylates and stabilizes PFKFB3, increasing F-2,6-BP levels and promoting glycolysis. However, under oxidative stress, reduced PFKFB3 methylation diverts the glucose flux toward the pentose phosphate pathway (PPP), generating NADPH and enhancing chemoresistance [191]. Similarly, CARM1 regulates amino acid and redox metabolism through the methylation of multiple substrates. It methylates PKM2 to suppress de novo serine synthesis [192] and methylates MDH1 at R230 to inhibit glutamine metabolism [193]. CARM1 also cooperates with NRF2 to upregulate G6PD and methylate RPIA at R42, sustaining NADPH production and redox homeostasis via PPP activation [194]. In addition, CARM1-mediated methylation of DRP1 at R403 and R634 promotes mitochondrial fission and contributes to redox signaling [188, 189].

#### Lipid metabolism and ferroptosis

PRMT5 is a major regulator of lipid metabolism and ferroptosis. It methylates SREBP1a, promoting de novo lipogenesis, a process further amplified by SIRT7-mediated desuccinylation [195]. In mantle cell lymphoma, PRMT5 promotes tumor growth by upregulating *SREBP1/2* and *FASN* expression through a *MYC*-dependent mechanism, thereby driving lipid metabolic reprogramming [196]. Beyond its role in lipid biosynthesis, PRMT5 also suppresses ferroptosis by stabilizing GPX4. PRMT5 catalyzes symmetric dimethylation of GPX4 at R152, which disrupts its interaction with the Cullin1–FBW7 E3 ubiquitin ligase complex, thereby preventing ubiquitination and proteasomal degradation [197]. In addition to PRMT5, CARM1 has also been implicated in ferroptosis regulation, where it functions as a negative regulator of ferroptotic cell death. Mechanistically, CARM1 has been shown to promote H3R26me2a deposition at the *GPX4* promoter, thereby sustaining GPX4 expression and suppressing lipid peroxidation [198]. Moreover, CARM1-mediated methylation of ACSL4 has been reported to limit ferroptosis sensitivity in colorectal cancer cells [199]. Through these mechanisms, CARM1 contributes to ferroptosis resistance by reinforcing antioxidant defenses and modulating lipid metabolic pathways. Collectively, these findings highlight the broader role of PRMT family members in coordinating lipid metabolic reprogramming and ferroptosis resistance in cancer.

#### Autophagy regulation

Autophagy represents a critical adaptive mechanism that enables cancer cells to survive metabolic and therapeutic stress. Emerging evidence indicates that PRMTs contribute to the fine-tuning of autophagy initiation and maturation. For instance, PRMT5 has been reported to methylate ULK1 at R170, thereby modulating autophagy initiation and stress responses [58]. In parallel, CARM1 regulates autophagy-related transcriptional programs through the AMPK–Skp2 axis. Under energy stress, AMPK activation promotes Skp2 degradation and subsequent stabilization of nuclear CARM1, which enhances the transcription of autophagy- and lysosome-related genes, in part through H3R17me2a deposition [45]. Moreover, CARM1-mediated methylation of Pontin is essential for the activation of this transcriptional program, facilitating the expression of genes required for autophagosome formation and lysosomal function [200]. Through these coordinated mechanisms, PRMTs integrate metabolic signaling with autophagy control, thereby contributing to tumor cell survival and therapeutic resistance.

#### Context-specific metabolic regulation (others)

Other PRMT family members exhibit tissue-specific and context-dependent metabolic functions. In lung cancer, PRMT6 regulates tumor metabolism by activating 6-phosphogluconate dehydrogenase (6PGD) and α-enolase (ENO1) through site-specific methylation. PRMT6 methylates 6PGD at R324 and increases its catalytic activity, enhancing oxidative PPP flux [201]. It also methylates ENO1 at R9 and R372, promoting active dimer formation and 2-phosphoglycerate binding, respectively, thereby stimulating glycolysis and tumor cell proliferation [201]. In HCC, PRMT6 methylates CRAF at R100, modulating ERK signaling and consequently regulating the nuclear translocation of PKM2, a key mediator of the Warburg effect. This PRMT6–ERK–PKM2 axis enhances glycolytic gene expression and contributes to tumorigenicity and drug resistance [202]. Conversely, loss of PRMT7 in chronic myeloid leukemia reprograms glycine metabolism, selectively eliminating leukemia stem cells [203].

Taken together, these findings establish PRMTs as multifaceted metabolic regulators that fine-tune the balance between energy production, biosynthesis, and redox homeostasis. PRMTs endow cancer cells with metabolic flexibility, which is essential for proliferation, survival, and therapeutic resistance, by coordinating glycolytic activation, amino acid metabolism, lipid synthesis, and ferroptosis resistance.

### Immunomodulation

Cancer immunity is a hallmark of tumor progression that influences how malignant cells evade immune surveillance and respond to therapy. Among the molecular regulators shaping these interactions, PRMTs have emerged as pivotal modulators that fine-tune immune signaling and determine the balance between immune activation and tolerance to cancer.

#### Antigen presentation and immune checkpoints

PRMTs reduce tumor immunogenicity by suppressing antigen presentation pathways, such as the STAT1–MHC-I signaling pathway. Using a CRISPR screen, PRMT1 was identified as a negative regulator of CD8^+^ T-cell-mediated cytotoxicity in melanoma. Mechanistically, PRMT1 suppresses *STAT1* transcription, reducing STAT1-driven MHC-I expression and attenuating CD8^+^ T-cell killing [204]. In the adaptive immune compartment, PRMT5 enhances the immunosuppressive activity of regulatory T cells resulting from FOXP3 methylation at R27, R51, and R126 [205]. PRMT5 also regulates long non-coding RNA genes encoding immunogenic micropeptides presented by MHC-I molecules and elicits potent CD8^+^ T-cell responses, adding a layer of tumor antigenicity [206].

In parallel, PRMTs upregulate immune checkpoint molecules, including PD-L1 and PD-L2, to further dampen T cell-mediated immunity. PRMT1 contributes to the upregulation of PD-L1 and PD-L2 in tumor cells by affecting promoter-linked expression and interferon signaling [207, 208]. PRMT5 promotes PD-L1 expression through H3R2me2s-mediated activation of *STAT1* transcription in cervical cancer [209], and H4R3me2s deposition at the *CD274* promoter in lung cancer [210]. Moreover, PRMT5 is upregulated by circGSK3β, a circular RNA derived from *GSK3B*, via miR-338-3p sponging, which in turn increases H3K4me3 at the *PD-L1* promoter, promoting immune evasion in breast cancer [211]. In HCC, PRMT3 promotes immune escape by upregulating PD-L1 via PDHK1-driven glycolysis. Specifically, PRMT3-mediated PDHK1 methylation at R363 and R368 enhances its kinase activity and increases lactate production. This lactate accumulation elevates H3K18la levels at the *PD-L1* promoter, further amplifying *PD-L1* transcription [212].

#### Innate immune signalling and inflammation

**Fig. 8:**
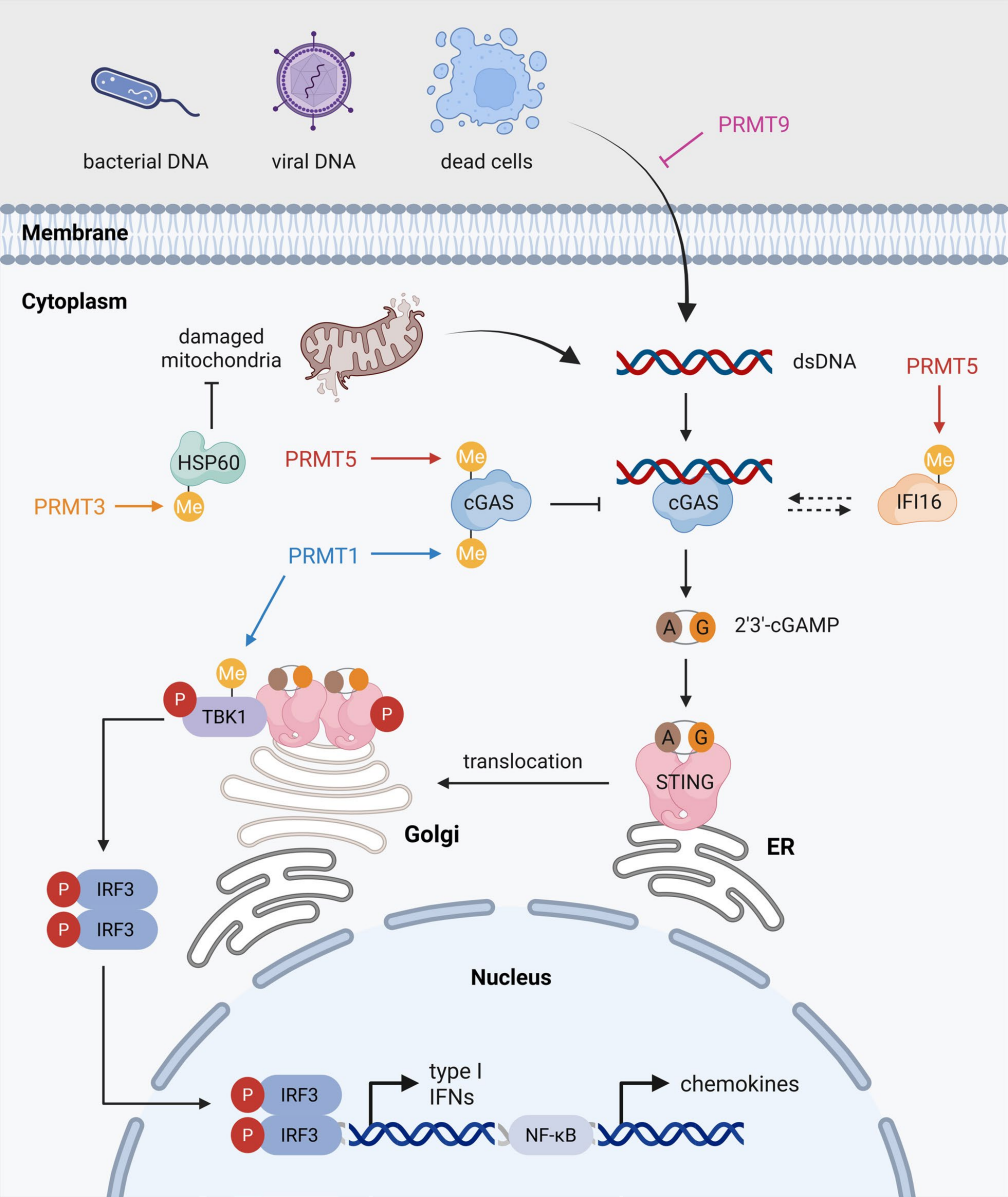
PRMT-mediated regulation of the cGAS/STING pathway. PRMT family members modulate innate immune signaling through methylation-dependent regulation of the cGAS–STING axis. PRMT1 (blue) methylates cGAS, attenuating cGAS activation and downstream STING signaling. PRMT3 (orange) methylates HSP60 to maintain mitochondrial integrity, whereas mitochondrial damage promotes mitochondrial DNA release into the cytosol and activation of cGAS. PRMT5 (red) methylates cGAS and IFI16, thereby regulating cytosolic DNA sensing. PRMT9 (pink) negatively regulates dsDNA-induced cGAS activation upstream of STING signaling. Yellow circles (Me) indicate methylation events, and red circles (P) represent phosphorylation. Solid arrows denote activation, whereas blunt-ended lines indicate inhibition

*cGAS/STING pathway:* Beyond immune checkpoint control, PRMTs orchestrate innate immune responses by methylating cytosolic DNA sensors and adaptor proteins, such as cGAS, STING, and NLRC5 (Fig. 8). The cGAS/STING pathway plays a critical role in detecting cytosolic DNA and initiating type I interferon responses, which influence nearly all aspects of tumorigenesis. Upon activation, cGAS/STING triggers TBK1 and IRF3 phosphorylation, leading to type I interferon-mediated antitumor immunity [213]. PRMT1 methylates cGAS at R133, preventing cGAS dimerization and suppressing cGAS/STING signaling. When PRMT1 is inhibited, the number of tumor-infiltrating lymphocytes increases in a cGAS-dependent manner [208]. Moreover, PRMT1 knockdown activates the cGAS/STING axis through enhanced dsDNA aggregation, increasing IFN-β secretion and shifting macrophages toward an M1-like phenotype [214]. In HCC, PRMT3 methylates HSP60 at R446 to promote oligomerization and maintain mitochondrial integrity. Consequently, PRMT3 inhibition induces mtDNA leakage, activating cGAS/STING–mediated antitumor immunity [215]. PRMT5 methylates IFI16, another component of the cGAS/STING pathway, attenuating DNA-induced interferon and chemokine production. It also represses *NLRC5* transcription, reduces MHC-I antigen presentation, and promotes immune evasion [216]. In AML and leukemia stem cells, PRMT9 inhibition disrupts RNA translation and DNA damage responses, activating cGAS/STING signaling, inducing type I interferon production, and promoting immunogenic cell death [217].

*RIG-I/MDA5 Pathway:* PRMTs regulate cytosolic RNA sensing via the RIG-I/MDA5/MAVS axis. PRMT7 maintains H4R3me2s-dependent silencing of endogenous retroviral elements (ERVs), and its loss derepresses ERVs, leading to dsRNA accumulation and activation of the RIG-I/MDA5/MAVS/TBK1/IRF3 pathway. This signaling cascade triggers type I interferon responses, enhancing antigen presentation and chemokine production [218]. Similarly, PRMT1 inhibition induced ERV re-expression and dsRNA accumulation, activating the RIG-I/MAVS pathway. The resulting interferon response increases PD-L1 expression, augments CD8^+^ T-cell infiltration, and synergizes with PD-1 blockade to enhance antitumor immunity [219].

#### Tumor microenvironment and immune cell modulation

PRMT-mediated methylation extends to the immune and stromal components of the tumor microenvironment, particularly macrophages and T cells. PRMT6 promotes lung tumor progression by driving M2-like polarization of tumor-associated macrophages (TAMs) and establishing an immunosuppressive tumor microenvironment through the PRMT6–ILF2–MIF axis [220]. Similarly, PRMT2 contributes to colorectal tumorigenesis by inducing M2-like TAM polarization and suppressing CD4^+^ and CD8^+^ T-cell activity [72]. PRMT1 exerts context-dependent antitumor effects. It sustains memory CD8^+^ T cell function via Wnt-driven reprogramming by depositing H4R3me2a at the *IL-2* promoter to maintain cytokine transcription and polyfunctional capacity [221]. In parallel, PRMT1 knockdown promotes M1-like macrophage polarization and reduces M2 infiltration both in vitro and in vivo, inhibiting tumor growth [214].

Collectively, PRMTs orchestrate multiple layers of immune regulation in cancer, including antigen presentation, innate immune sensing, inflammatory signaling, and tumor microenvironment remodeling. By selectively modulating these pathways, PRMTs can either promote antitumor immune activation or facilitate immune evasion. Their dual ability to suppress immune visibility, while simultaneously triggering interferon-driven cytotoxicity, highlights the context-dependent nature of arginine methylation in tumor immunity. These multifaceted roles position PRMTs as central epigenetic regulators at the interface of oncogenic signaling and the immune response, offering a compelling rationale for targeting PRMTs to enhance immunotherapy efficacy.

## PRMT inhibitors and therapeutic targeting

PRMTs have emerged as attractive targets for cancer therapy because of their central roles in epigenetic remodeling, transcriptional regulation, metabolic reprogramming, immune modulation, and metastatic progression [5, 31]. The development of small-molecule PRMT inhibitors has advanced rapidly, demonstrating promising efficacy in preclinical models. However, clinical translation has been limited by challenges related to selectivity, toxicity, and pharmacokinetics. This section summarizes the PRMT inhibitors currently under preclinical (Table 2) and clinical development (Table 3), and outlines recent advances in PRMT-targeting strategies, including proteolysis-targeting chimera (PROTAC)-based degraders and synthetic lethality-guided precision therapies.

**Table 2:** PRMT inhibitors

Target	Compound	Chemical Structure	IC_50_	MOA	Ref
Type I PRMTs	AMI-1		8.8 μM	substrate pocket binding	[299]
	MS023		PRMT1 (30 nM) PRMT3 (119 nM) CARM1 (83 nM) PRMT6 (4 nM) PRMT8 (5 nM)	allosteric	[300]
	EPZ019997 (GSK3368715)		PRMT1 (33.1 nM) PRMT3 (162 nM) CARM1 (38 nM) PRMT6 (4.7 nM) PRMT8 (3.9 nM)	bind near active site, affecting substrate and SAM binding region	[241]
PRMT1	DCP1061		PRMT1 (5 nM) PRMT6 (5 nM) PRMT8 (5 nM)	bind near active site, affecting substrate and SAM binding region	[265]
	TC-E-5003		1.5 μM	substrate pocket binding	[301]
PRMT3	SGC707		31 nM	allosteric	[302]
CARM1	TP-064		CARM1 (10 nM) PRMT6 (1.3 μM) PRMT8 (8.1 μM)	substrate pocket binding	[224]
	EZM2302 (GSK3359088)		6 nM	substrate pocket binding	[226]
	EPZ0025654 (GSK35336023)		3 nM	-	[274]
	SKI-73		1.3 μM	substrate pocket binding	[227]
	YD1342 (prodrug)		< 1 nM	substrate pocket binding	[303]
	SGC2085		50 nM	substrate pocket binding	[304]
CARM1 PRMT6	MS049		CARM1 (34 nM) PRMT6 (43 nM)	substrate pocket binding	[300]
PRMT6	EPZ020411		10 nM	substrate pocket binding	[305]
	SGC6870		77 nM	allosteric	[306]
	MS117		18 nM	active site binding	[307]
	GMS		90 nM	active site binding	[308]
PRMT5	EPZ015938 (GSK3326595) (pemrametostat)		189 – 237 nM	SAM-cooperative, substrate pocket binding	[98]
	EPZ015666 (GSK3235025)		22 nM	substrate pocket binding	[309]
	EPZ015866 (GSK591, GSK320291)		4 nM	-	[310]
	JNJ-64619178 (Onametostat)		0.14 nM	SAM-competitive	[311]
	LLY-283		22 nM	SAM-competitive	[229]
	PF-06939999		1.1 nM	SAM-competitive	[312]
	PF-06855800		1 nM	SAM-competitive	[313]
	PRT543		10.8 nM	SAM-competitive	[314]
	PRT811		3.9 nM	SAM-competitive	[315]
	AM-9747		0.06 nM	substrate pocket binding	[316]
	TNG462 (Vopimetostat)		-	MTA cooperative, substrate pocket binding	[317]
	TNG456		-	MTA cooperative, substrate pocket binding	[318]
	TNG908 (Ralometostat)		21.2 nM	MTA cooperative, substrate pocket binding	[319]
	AMG-193		107 nM (MTAP-deleted cell)	MTA cooperative, substrate pocket binding	[254]
	BMS-986504 MRTX1719 (Navlimetostat)		3.6 nM	MTA cooperative, substrate pocket binding	[255]
	MRTX9768		11 nM	MTA cooperative, substrate pocket binding	[320]
	AZD3470		-	MTA cooperative	[256]
	BGB-58067	Unveiled	-	MTA cooperative	NCT06589596
	BAY3713372	Unveiled	-	MTA cooperative	NCT06914128
PRMT5 PRMT9	MRK-990		PRMT5 (10 nM) PRMT9 (30 nM)	-	[321]
PRMT9	EML1219		0.2 μM	substrate pocket binding	[322]
	LD2		2–7 μM	catalytic pocket binding	[217]
PRMT7	SGC3027 (prodrug of SGC8158)		294 nM	SAM-competitive	[230]
	SGC8158		2–9 μM	SAM-competitive	[231]
	JS1310		PRMT7 (5 μM) PRMT5 (50 μM)	-	[203]
PRMT5 PRMT7	DS-437		PRMT5 (5.9 μM) PRMT7 (6 μM)	SAM-competitive	[323]
Pan-inhibitor	DB75 (Furamidine)		PRMT1 (9.4 μM) PRMT5 (166 μM) PRMT6 (283 μM) CARM1 (> 400 μM)	SAM-competitive	[324]

**Table 3:** PRMT Inhibitors in Clinical Trials

Target	Compound	Clinical trial (NCT)	Phase	Treatment type	Indication	Status
Type I PRMTs	EPZ019997 (GSK3368715)	NCT03666988	Phase 1	Single agent	Advanced solid tumors, DLBCL	Terminated (Early)
PRMT5	EPZ015938 (GSK3326595) (Pemrametostat)	NCT02783300 (Meteor 1)	Phase 1	GSK3326595 ± pembrolizumab	Selected solid tumors; part cohorts included NHL/NSCLC etc	Completed
		NCT03614728	Phase 1/2	GSK3326595 ± 5-azacitidine	MDS / AML	Terminated
		NCT04676516	Phase 2	Single agent	Early-stage HR-positive breast cancer	Completed
PRMT5	JNJ-64619178 (Onametostat)	NCT03573310	Phase 1	Single agent	Advanced solid tumors, NHL, lower-risk MDS	Active, not recruiting
		NCT06788509	Phase 1	Single agent	AML, NHL, MDS, CLL, Advanced solid tumors and mCRPC	Enrolling by invitation
PRMT5	PF-06939999	NCT03854227	Phase 1	PF-06939999 ± docetaxel	Advanced/metastatic solid tumors	Terminated
PRMT5	PRT543	NCT03886831	Phase 1	Single agent	Advanced Solid Tumors and Hematologic Malignancies	Completed
PRMT5	PRT811	NCT04089449	Phase 1	Single agent	Advanced Solid Tumors, CNS Lymphoma and Gliomas	Completed
PRMT5	TNG462 (Vopimetostat)	NCT05732831	Phase 1/2	TNG462 ± pembrolizumab	MTAP-deleted solid tumors	Recruiting / Active
		NCT06188702	Phase 1/2	TNG462 ± MAT2A inhibitor	Advanced or metastatic solid tumors with deletion of MTAP	Recruiting / Active
		NCT06922591	Phase 1/2	TNG462 ± RAS inhibitors	PDAC and NSCLC	Recruiting / Active
PRMT5	TNG456	NCT06810544	Phase 1/2	TNG456 ± abemaciclib	MTAP-deleted solid tumors	Recruiting / Active
PRMT5	TNG908 (Ralometostat)	NCT05275478	Phase 1/2	Single agent	MTAP-deleted Solid Tumors	Active, not recruiting
PRMT5	AMG-193	NCT06333951 (MTAPESTRY104)	Phase 1	AMG-193 ± carboplatin/paclitaxel/pembrolizumab/pemetrexed/sotorasib	Advanced thoracic tumors with homozygous MTAP deletion (master protocol)	Active / Recruiting
		NCT05094336 (MTAPESTRY101)	Phase 1/2	AMG-193 ± docetaxel	Advanced MTAP-null solid tumors	Recruiting / Active
		NCT06593522	Phase 2	Single agent	MTAP-deleted advanced NSCLC	Active / Recruiting
		NCT06360354 (MTAPESTRY103)	Phase 1	AMG-193 ± gemcitabine/nab-paclitaxel/mFOLFIRINOX	Advanced GI / biliary / pancreatic cancers with MTAP deletion	Recruiting / Active
		NCT05975073	Phase 1/2	AMG-193 ± IDE397	Advanced MTAP-null Solid tumors	Active, not recruiting
PRMT5	BMS-986504 / MRTX1719 (Navlimetostat)	NCT05245500	Phase 1	Single agent	MTAP-deleted solid tumors	Active / Recruiting
		NCT06883747	Phase 0/1	Single agent	MTAP-deleted recurrent GBM	Recruiting
		NCT07077434	Phase 1	Single agent	Advanced Solid Tumors	Active / Not yet recruiting
		NCT07076121 (MountainTAP-30)	Phase 2/3	BMS-986504 ± gemcitabine/nab-paclitaxel	Untreated Metastatic PDAC with Homozygous MTAP Deletion	Active / Recruiting
		NCT06855771 (MountainTAP-9)	Phase 2	Single agent	Advanced or metastatic NSCLC with homozygous MTAP deletion (refractory)	Recruiting
		NCT06672523	Phase 1	Single agent	Advanced Solid Tumors with Homozygous MTAP Deletion	Recruiting
		NCT07063745 (MountainTAP-29)	Phase 2/3	BMS-986504 ± pembrolizumab/platinum agent/pemetrexed/paclitaxel	First-line Metastatic Non-small Cell Lung Cancer Participants with Homozygous MTAP Deletion	Recruiting
PRMT5	AZD3470	NCT06130553 (PRIMROSE)	Phase 1/2	Single agent	MTAP-deficient advanced/metastatic solid tumors	Recruiting
		NCT06137144	Phase 1/2	AZD3470 ± combos	Relapsed/refractory hematologic malignancies	Recruiting / active
PRMT5	BGB-58067	NCT06589596	Phase 1	BGB-58067 ± BG-89894	Advanced solid tumors with MTAP deletion	Recruiting / Active
PRMT5	BAY3713372	NCT06914128	Phase 1/2	Single agent	MTAP-deleted Solid Tumors	Recruiting

*AML* Acute Myeloid Leukemia, *CLL* Chronic Lymphocytic Leukemia *DLBCL* Diffuse Large B-Cell Lymphoma, *GBM* Glioblastoma Multiforme HR-positive breast cancer; hormone receptor-positive breast cancer, *MDS* myelodysplastic syndromes, *mCRPC* Metastatic Castration-Resistant Prostate Cancer, *MTAP* Methylthioadenosine Phosphorylase, *NHL* Non-Hodgkin Lymphoma, *NSCLC* Non-Small Cell Lung Cancer, *PDAC* Pancreatic Ductal Adenocarcinoma

### Preclinical studies

Broad-spectrum inhibitors, such as AMI-1 and MS023, target type I PRMTs and have demonstrated anti-metastatic effects in several cancer models. In breast cancer, AMI-1 increases p16 and p21 expression by inhibiting PRMT1-mediated EZH2 methylation, reducing cell proliferation and metastasis [90, 222]. MS023 also decreases metastasis by suppressing oncogene expression and enhancing cytotoxic T-cell infiltration through the activation of interferon antiviral response pathways [223].

Selective inhibitors improve specificity and reduce off-target effects. SGC707 selectively inhibits PRMT3 and disrupts LDHA methylation and glycolytic reprogramming in hepatocellular carcinoma [182, 212]. CARM1 inhibitors, including TP-064 [224, 225], EZM2302 (GSK3359088) [226], and SKI-73 [227], suppress BAF155 methylation, leading to reduced recruitment of BRD4 to super-enhancers and subsequent downregulation of oncogenes such as *MYC*. These inhibitors also enhance interferon signaling and increase CD8^+^ T cell infiltration, reducing the metastatic potential, particularly in breast cancer models. EPZ020411*,* a PRMT6-selective inhibitor, blocks R729 methylation and subsequent Y705 phosphorylation of STAT3, ultimately suppressing its metastatic dissemination [169].

Preclinical studies have demonstrated that PRMT5 inhibitors effectively suppress EMT and metastatic progression in multiple cancer models. For example, EPZ015666 (GSK3235025) inhibits PRMT5-mediated formation of the Slug-LSD1 complex in breast cancer, restores E-cadherin expression, and reduces lung metastasis [162]. In head and neck squamous cell carcinoma, EPZ015666 disrupts the PRMT5/WDR5-dependent H3R2me2s-H3K4me3 axis, suppressing *TWIST1* transcription and lymph node metastasis [228]. In cervical cancer, EPZ015666 interferes with the Snail/PRMT5/NuRD complex, restoring TET1 expression and increasing 5-hydroxymethylcytosine levels, thereby attenuating EMT and invasion [161]. Other PRMT5 inhibitors also show anti-metastatic effects: LLY-283 reduces proliferation and metastasis in head and neck squamous cell carcinoma [229]. EPZ015938 inhibits EMT-related phenotypes by disrupting the ZEB2/Twist1/PRMT5/NuRD complex that epigenetically represses E-cadherin expression in colorectal cancer [163].

PRMT7 inhibitors, including SGC3027, SGC8158, and JS1310, have been developed and investigated [230, 231]; however, further optimization is required in terms of selectivity, bioavailability, and safety for translational applications.

### Clinical trials

The clinical development of PRMT inhibitors is largely limited to early-phase studies, primarily focusing on assessing their safety, pharmacokinetics, pharmacodynamics, and preliminary efficacy. The current status of clinical trials investigating PRMT inhibitors is summarized in Table 3.

Clinical progress of type I PRMT inhibitors is limited. GSK3368715, a potent SAM-noncompetitive inhibitor, demonstrated broad preclinical efficacy but was discontinued in Phase I trials (NCT03666988) owing to thromboembolic toxicity and insufficient efficacy, underscoring the need for safer and more selective compounds. Instead, PRMT5 inhibitors have advanced further clinically, especially in MTAP-deficient tumors, exploiting synthetic lethality to enhance the selectivity and therapeutic index. EPZ015938 has been evaluated as a monotherapy (NCT04676516), in combination with pembrolizumab for selected tumors (NCT02783300), and with 5-azacitidine for relapsed/refractory myelodysplastic syndrome and AML (NCT03614728). PF-06939999 was evaluated in advanced/metastatic solid tumors, both as a monotherapy and combined with docetaxel (NCT03854227). JNJ-64619178 has completed a Phase I evaluation for advanced solid tumors, non-Hodgkin lymphoma, and lower-risk myelodysplastic syndrome (NCT03573310). Moreover, next-generation MTAP-selective or MTA cooperative inhibitors, including AZD3470, AMG-193, BGB-58067, MRTX1719, TNG456, and TNG462 are being tested in MTAP-deleted advanced solid tumors, either alone or in combination with standard chemotherapy, CDK4/6 inhibitors or immune checkpoint inhibitors (Table 3). Many of these agents remain in early dose escalation and expansion cohorts.

These clinical studies indicate that PRMT inhibitor development focuses on early phase evaluation in genetically defined populations, with combination therapy strategies emerging as the principal approach to maximize the therapeutic potential. The establishment of early safety, pharmacokinetic/pharmacodynamic profiles, and preliminary antitumor activity provides a strong rationale for continued and systemic clinical investigation of PRMT inhibitors.

### PROTACs

PRMTs exert not only critical enzymatic functions but also essential scaffold roles that influence oncogenesis and other cellular processes; therefore, the development of targeted protein degradation technologies, such as PROTACs, has accelerated to enable more complete functional suppression. Recent advances have enabled the generation of PROTACs that target multiple members of the PRMT family.

For PRMT1, both CRBN- and VHL-recruiting degraders have been reported to achieve moderate degradation efficiencies [232]. These early molecules revealed key structural determinants and offer a foundation for further optimization of linker architecture and E3 ligase selection. A selective PRMT3 degrader using an MDM2-based PROTAC design effectively reduced PRMT3 protein levels, leading to the suppression of ADMA marks [233]. It exhibited superior anti-leukemic efficacy compared to the catalytic inhibitor SGC707 by inducing apoptosis and endoplasmic reticulum stress in leukemia cells. For CARM1, both VHL-based CARM1 degrader-1 (derived from TP-064) and C199 (derived from EZM2302) demonstrated robust and selective degradation [234, 235]. CARM1 degrader-1 potently depleted CARM1 (DC_50_ = 8 nM; D_max_ > 95%) and effectively inhibited breast cancer cell migration. C199 also exhibited strong degradation activity (DC_50_ = 106 nM, D_max_ = 93.1%) and favorable pharmacokinetics. In vivo, C199 induced near-complete CARM1 depletion and markedly inhibited tumor growth (TGI = 78%).

Among the PRMT family members, PRMT5-targeting PROTACs are the most advanced. The first-generation VHL-based degrader MS4322 achieved moderate degradation (DC_50_ = 1,100 nM; D_max_ = 74%), but displayed relatively slow kinetics [236]. The optimized derivative MS115, which is also VHL-based, exhibited improved potency and induced rapid proteasome-dependent degradation of both PRMT5 and its cofactor MEP50 in breast and prostate cancer models [237]. CRBN-recruiting degraders have further enhanced efficacy; YZ-836P demonstrates potent activity with a DC_50_ of approximately 10 nM and > 80% maximal degradation, suppressing tumor growth in TNBC organoids and xenografts [238].

### Therapeutic implications

PRMT inhibitors exert multifaceted antitumor effects by modulating the chromatin architecture, transcriptional programs, metabolic flux, immune evasion, and metastatic potential. Although extensive preclinical data underscore their promise, their clinical efficacy has been tempered by toxicity, limited selectivity, and pharmacokinetic challenges. To overcome these barriers, current therapeutic strategies increasingly emphasize rational drug combinations and the exploitation of context-specific vulnerabilities, such as synthetic lethality.

#### Combination therapy

While single-agent PRMT inhibition has shown potent activity in preclinical settings, clinical responses have been modest. Accordingly, combination strategies have emerged as major strategies for enhancing efficacy and selectivity. These approaches are broadly categorized into (i) exploiting PRMT-driven vulnerabilities such as DNA damage and splicing stress, (ii) reversing PRMT-mediated immune suppression, and (iii) targeting compensatory oncogenic signaling pathways that limit the efficacy of PRMT inhibitors.

First, combinations of DNA damage- or splicing-targeting agents have a strong mechanistic rationale. Both PRMT1 and PRMT5 are integral to the DDR and replication stress responses. Their inhibition sensitizes tumors to PARP1, ATR, and Chk1 inhibitors as well as DNA-damaging chemotherapies [239, 240]. Notably, PRMT5 inhibition induced robust synthetic lethality when used in combination with PARP1 inhibition [239, 240]. In addition, dual blockade of PRMT1 and PRMT5 synergistically suppresses tumor growth by causing profound splicing defects [241].

Second, immuno-oncology combinations represent a translationally advanced frontier. PRMT5 inhibition reactivates interferon signaling, enhances antigen presentation, and promotes T-cell infiltration by reversing epigenetically silenced immune pathways [209]. Consequently, PRMT5 inhibitors have shown synergy with the PD-1/PD-L1 immune checkpoint blockade in several tumor models, leading to their incorporation into early phase clinical trials (NCT02783300, NCT06333951). Early observations from these studies further suggested enhanced remodeling of the tumor microenvironment and more durable antitumor immune responses, supporting the rationale for continued clinical development of these combination strategies.

Third, targeted signaling co-dependency provides an additional layer of therapeutic precision. PRMT5 regulates the components of the PI3K/Akt and CDK pathways, suggesting that combined inhibition can yield additive or synergistic tumor suppression [119, 242, 243]. Similarly, type I PRMT inhibitors sensitize EGFR- or c-Myc-dependent cancers to targeted kinase inhibitors and BET inhibitors, effectively co-disabling upstream drivers and downstream effectors [244, 245].

#### Synthetic lethality

Synthetic lethality occurs when the simultaneous loss of two genes leads to cell death, whereas the loss of either gene alone is associated with cell survival. This principle provides a powerful strategy to selectively eliminate tumor cells harboring specific mutations, while sparing normal tissues [246]. MTAP is an enzyme that degrades MTA, a byproduct of polyamine metabolism, to produce adenine and methylthioribose-1-phosphate for the methionine salvage pathway. MTAP deletion is a genetic alteration frequently co-occurring with *CDKN2A* loss in various cancers, leading to the abnormal accumulation of intracellular MTA [247]. The accumulated MTA competes with SAM in the catalytic pocket of PRMT5, partially inhibiting PRMT5 activity (Fig. 2). Therefore, MTAP-deleted tumors exhibit an abnormal dependence on PRMT5, and this “hypomorphic PRMT5” state renders the cancer cells exquisitely sensitive to further pharmacologic inhibition of PRMT5. This provides a mechanistic basis for exploiting MTAP deletion as a selective anticancer therapeutic strategy [248].

Methionine adenosyltransferase 2A (MAT2A) catalyzes the synthesis of SAM. Inhibition of MAT2A reduces SAM pools, limiting substrate availability for PRMT5. Although MAT2A inhibition alone is only modestly cytostatic, it strongly synergizes with PRMT5 blockade in MTAP-deficient cells, in which PRMT5 function is compromised by MTA accumulation [249]. Together, this dual inhibition perturbs the methionine-SAM methylation axis, leading to SDMA depletion, splicing defects, and apoptosis, while sparing MTAP-proficient cells that maintain normal methylation capacity [249]. To capitalize on this metabolic vulnerability, next-generation MTA cooperative PRMT5 inhibitors, such as TNG462, TNG908, and MRTX1719, have been engineered to bind preferentially to the PRMT5–MTA complex. These agents exhibited enhanced potency under MTA-rich and MTAP-deleted conditions, while maintaining reduced activity in MTAP-proficient cells. This design provides a refined tumor-selective pharmacological window that links metabolic derangements to epigenetic inhibition [250].

Emerging evidence suggests that PRMT1 may represent a context-dependent synthetic lethality target beyond the canonical MTAP–PRMT5 axis. In VHL-deficient ccRCC, PRMT1 functions as a critical tumor dependency, and its inhibition induces cell cycle arrest, widespread disruption of mRNA metabolism, R-loop accumulation, and DNA damage, thereby impairing tumor growth [108]. In EGFR- and KRAS-mutant lung cancer models, PRMT1 promotes cancer cell persistence during targeted therapy, and its genetic or pharmacologic inhibition enhances the efficacy of EGFR or KRAS inhibitors, reduces tumor regrowth, and prolongs tumor regression in vivo [244]. Furthermore, in contexts of low PRMT5 expression or compromised PRMT5 activity, tumor cells may exhibit compensatory reliance on PRMT1-mediated asymmetric arginine methylation to sustain transcriptional programs, RNA processing, and proteostasis [251]. Collectively, these findings support the notion that PRMT1 dependency can emerge in defined genetic and signaling contexts, highlighting its potential as an alternative or complementary synthetic lethality–based therapeutic target and broadening the scope of PRMT-directed precision oncology.

## Challenges and future perspectives

PRMTs have emerged as pivotal regulators of gene expression, metabolism, and cellular signaling, making them attractive targets for cancer therapy. Despite their strong biological validation and promising preclinical efficacy, their translation into durable clinical success has been limited by several challenges. Even inhibitors targeting the same enzyme may exhibit distinct substrate specificities in various cellular contexts. For example, TP-064 and EZM2302 have distinct mechanisms for modulating CARM1 substrates and downstream pathways [252]. This substrate-selective inhibition has important implications for both experimental design and therapeutic development, underscoring the necessity for context-specific selection of CARM1 inhibitors in basic research and precision oncology. The major barriers to effective clinical application include pharmacological limitations, adaptive tumor responses, patient stratification, and the inherent complexity of PRMT biology, which extends beyond enzymatic catalysis.

PRMTs are essential for normal cellular functions, including hematopoiesis, RNA processing, and chromatin maintenance. Systemic inhibition has, therefore, revealed a narrow therapeutic index: type I PRMT inhibitors (GSK3368715) cause thromboembolic events [253] and PRMT5 inhibitors (EPZ015938 and PF-06939999) exhibit hematologic dose-limiting toxicities. These findings underscore the need for tumor-targeted delivery or context-dependent modulation to enhance tolerability.

Cancer cells can evade PRMT inhibition via compensatory mechanisms, including the upregulation of alternative methyltransferases, metabolic rewiring, or epigenetic adaptation. Functional overlap among PRMT isoforms further complicates sustained inhibition. Therefore, rational combination strategies, involving DDR modulators, immune checkpoint inhibitors, or kinase-targeted therapies, are essential to overcome drug resistance and improve efficacy.

Biomarker-guided patient stratification is an urgent requirement for successful clinical translation. MTAP deletion is currently the most established predictive biomarker, enabling the synthetic lethality-based targeting of PRMT5 through MTA cooperative or MTAP-selective inhibitors such as AMG-193, MRTX1719, and AZD3470 [254–256]. However, reliable biomarkers for the other PRMT isoforms are lacking. Integrative multiomics profiling, including methylation signatures, splicing dependency patterns, and immunomodulatory phenotypes, is expected to identify new responder subsets and guide the precise application of PRMT-targeted therapies.

Many PRMTs possess scaffolding or regulatory roles that are independent of their catalytic activity [5, 31, 173]. Consequently, enzymatic inhibition alone may not abrogate oncogenic functions. Targeted protein degradation strategies such as PROTACs allow the simultaneous elimination of both catalytic and structural functions. Early PROTACs targeting PRMT5 and CARM1 demonstrated potent and selective degradation and enhanced phenotypic effects, although optimization of pharmacokinetics and in vivo efficacy is ongoing.

In conclusion, advancing PRMT-targeted therapy will require the convergence of two strategic directions: (i) biomarker-based precision medicine to define responsive patient populations (e.g., MTAP deletion, methylation or splicing dependencies) and (ii) next-generation modalities, including isoform-selective inhibitors, antisense oligonucleotides, and targeted degraders, to achieve potent, selective, and durable tumor suppression with minimal systemic toxicity. With these integrated approaches, the field is poised to transition from proof-of-concept inhibition to clinically meaningful and long-lasting control of PRMT-driven malignancies.

## Data Availability

No datasets were generated or analysed during the current study.
